# Development of a new class of PSMA radioligands comprising ibuprofen as an albumin-binding entity

**DOI:** 10.7150/thno.40482

**Published:** 2020-01-01

**Authors:** Luisa M. Deberle, Martina Benešová, Christoph A. Umbricht, Francesca Borgna, Manuel Büchler, Konstantin Zhernosekov, Roger Schibli, Cristina Müller

**Affiliations:** 1 Department of Chemistry and Applied Biosciences, ETH Zurich, 8093 Zurich, Switzerland.; 2 Center for Radiopharmaceutical Sciences ETH-PSI-USZ, Paul Scherrer Institute, 5232 Villigen-PSI, Switzerland.; 3 Isotope Technologies Garching GmbH, 85748 Garching, Germany.

**Keywords:** prostate cancer, PSMA ligands, ^177^Lu, albumin-binder, ibuprofen.

## Abstract

Prostate-specific membrane antigen (PSMA)-targeted radioligands have been used for the treatment of metastatic castration-resistant prostate cancer (mCRPC). Recently, albumin-binding PSMA radioligands with enhanced blood circulation were developed to increase the tumor accumulation of activity. The present study aimed at the design, synthesis and preclinical evaluation of a novel class of PSMA-targeting radioligands equipped with ibuprofen as a weak albumin-binding entity in order to improve the pharmacokinetic properties.

**Methods**: Four novel glutamate-urea-based PSMA ligands were synthesized with ibuprofen, conjugated via variable amino acid-based linker entities. The albumin-binding properties of the ^177^Lu-labeled PSMA ligands were tested *in vitro* using mouse and human plasma. Affinity of the radioligands to PSMA and cellular uptake and internalization was investigated using PSMA-positive PC-3 PIP and PSMA-negative PC-3 flu tumor cells. The tissue distribution profile of the radioligands was assessed in biodistribution and imaging studies using PC-3 PIP/flu tumor-bearing nude mice.

**Results**: The PSMA ligands were obtained in moderate yields at high purity (>99%). ^177^Lu-labeling of the ligands was achieved at up to 100 MBq/nmol with >96% radiochemical purity. *In vitro* assays confirmed high binding of all radioligands to mouse and human plasma proteins and specific uptake and internalization into PSMA-positive PC-3 PIP tumor cells. Biodistribution studies and SPECT/CT scans revealed high accumulation in PC-3 PIP tumors but negligible uptake in PC-3 flu tumor xenografts as well as rapid clearance of activity from background organs and tissues. ^177^Lu-Ibu-DAB-PSMA, in which ibuprofen was conjugated via a positively-charged diaminobutyric acid (DAB) entity, showed distinguished tumor uptake and the most favorable tumor-to-blood and tumor-to-kidney ratios.

**Conclusion**: The high accumulation of activity in the tumor and fast clearance from background organs was a common favorable characteristic of PSMA radioligands modified with ibuprofen as albumin-binding entity. ^177^Lu-Ibu-DAB-PSMA emerged as the most promising candidate; hence, more detailed preclinical investigations with this radioligand are warranted in view of a clinical translation.

## Introduction

Prostate cancer is one of the most prevalent cancer types in men and the third leading cause of cancer-related death in the western world 1, 2. The treatment of metastatic castration-resistant prostate cancer (mCRPC) remains challenging and options to cure patients at this stage of the disease are currently not available. The developments of new concepts for more effective therapies are, therefore, urgently needed.

The prostate-specific membrane antigen (PSMA) is a transmembrane glycoprotein, which is overexpressed in approximately 90% of prostate cancer cases 3-6. As a result, it has been identified as a promising target for nuclear imaging of prostate cancer and for targeted radionuclide therapy of mCRPC 7-9. Various PSMA-targeting radioligands were developed in recent years, whereof ^68^Ga-PSMA-11 has emerged as the current “gold standard” for PET imaging of PCa patients with biochemical recurrence and for re-staging of the disease after therapy 10-13. In view of a therapeutic application, the investigations were focused on the impact of variable linker entities and chelators on the radioligands' pharmacokinetic properties 14, 15. Among the most promising radioligands were PSMA-617 14 and PSMA I&T 16 with a DOTA- and DOTAGA-chelator, respectively. These ligands allow the application of a number of radionuclides, including ^177^Lu (T_1/2_ = 6.65 d; Eβ^-^_av_ = 134 keV, Eγ = 113 keV; 208 keV), which is employed for PSMA-targeted radionuclide therapy of mCRPC.

The concept of modifying radiopharmaceuticals with an albumin-binding entity, originally demonstrated with a folate radioconjugate 17, has also been applied to PSMA-targeting radioligands 18. The *p*-iodophenyl moiety, previously identified as a strong albumin-binding entity 19, and Evans blue (EB), an azo dye with high affinity to albumin 20, 21, have been used for this purpose. Several preclinical studies revealed increased tumor uptake and, hence, better therapeutic efficacy of PSMA radioligands derivatized with the *p*-iodophenyl moiety as compared to the non-albumin binding reference radioligands 22-25. Wang et al. were the first to use EB for the derivatization of a PSMA radioligand 26 including the development of EB-derivatized PSMA-617 27. Based on the improved therapeutic efficacy of ^177^Lu-EB-PSMA-617 in mice as compared to ^177^Lu-PSMA-617 27, ^177^Lu-EB-PSMA-617 was translated to a first-in-human clinical study, which showed promising results 28.

The benefit of an enhanced tumor uptake of long-circulating PSMA radioligands is, however, compromised by an increased retention of activity in healthy organs and tissues including the kidneys and bone marrow, which may limit the number of therapy cycles that can be applied. Albumin-binding properties have, thus, to be carefully balanced to achieve an increased tumor uptake while keeping background activity as low as possible. In recent studies of our group, the *p*-iodophenyl-based albumin binder of ^177^Lu-PSMA-ALB-53 was, therefore, replaced by a the *p*-tolyl entity as a weaker albumin binder 19 in order to obtain ^177^Lu-PSMA-ALB-56 29. This radioligand resulted in more favorable tumor-to-background ratios and revealed enhanced therapeutic efficacy, however, tumor-to-blood ratios were still high, possibly even limiting in view of a therapeutic application.

**Figure 1:**
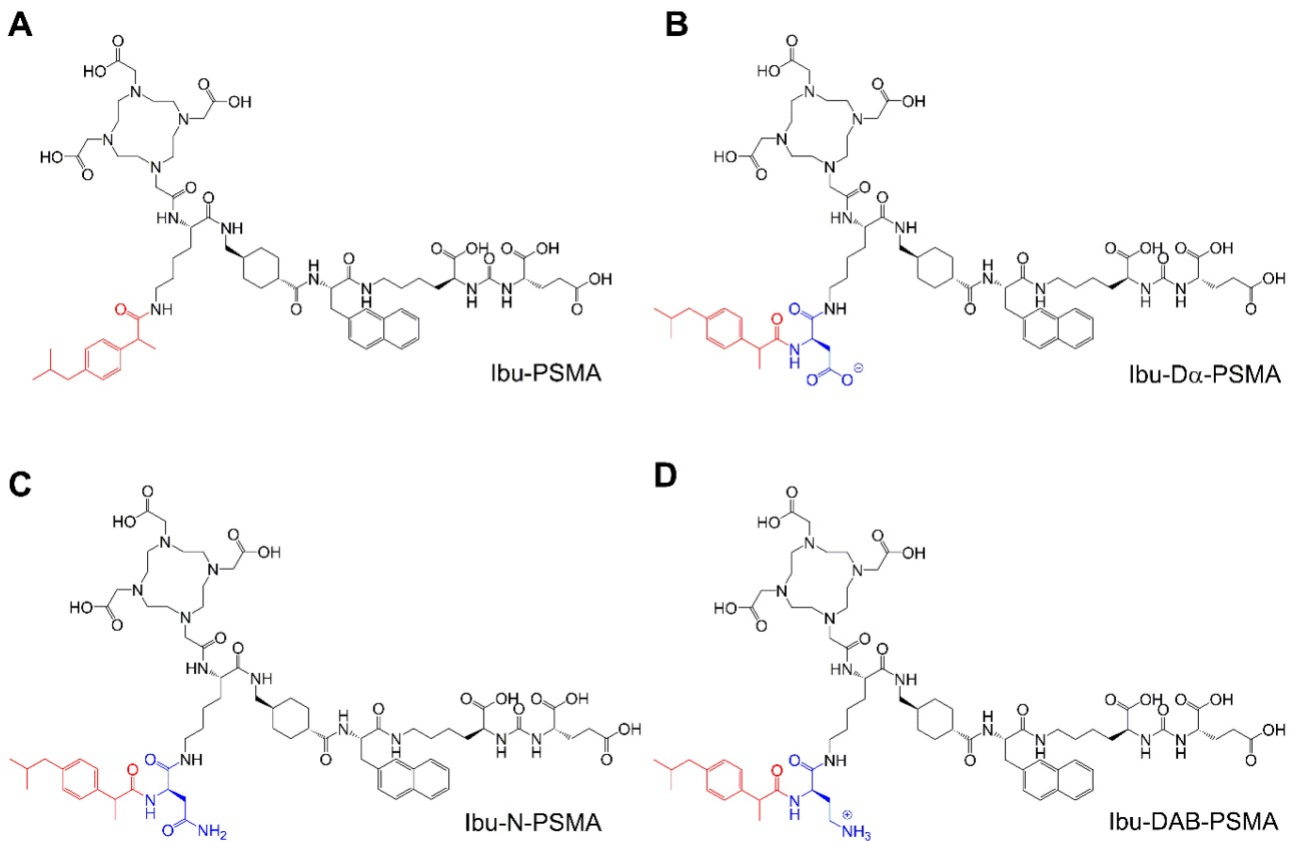
Chemical structures of the ibuprofen-derivatized, albumin-binding PSMA ligands. (A) Ibu-PSMA, (B) Ibu-Dα-PSMA, (C) Ibu-N-PSMA and (D) Ibu-DAB-PSMA.

The aim of this study was, therefore, to use a different albumin-binding entity in order to reach an optimum compromise between plasma protein-binding properties to achieve high tumor uptake, but efficient clearance from the blood pool to keep background activity in healthy organs and tissues as low as possible. The isobutylphenyl propionic acid, known under the name “ibuprofen”, is a non-steroidal anti-inflammatory drug (NSAID), which binds to plasma proteins 30, 31. This molecule was selected for modification of the PSMA ligands by conjugation using amino acids with differently charged side chains (Figure 1). The radioligands were evaluated in a preclinical setting in order to determine the most promising candidate.

## Methods

### Synthesis of ibuprofen-derivatized PSMA ligands

**Scheme 1:**
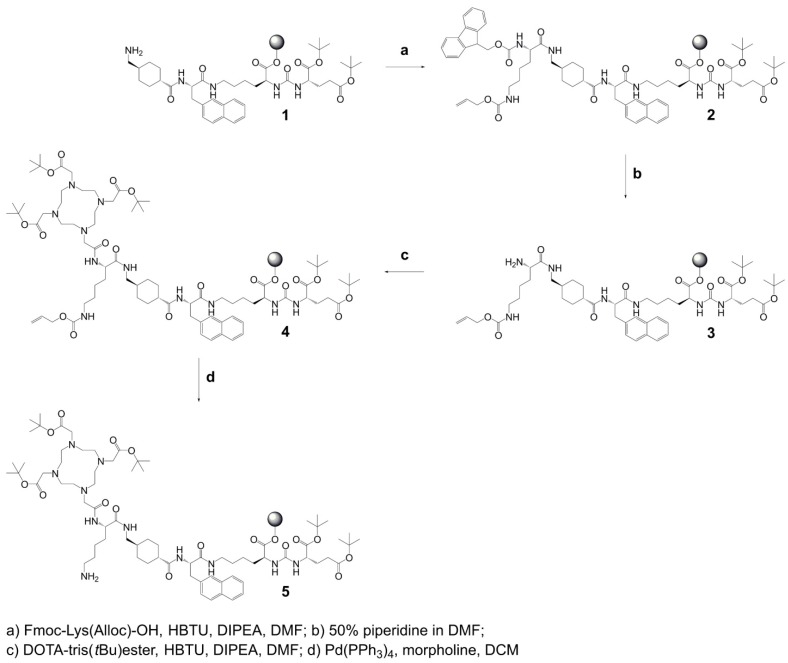
Synthesis of joint precursor 5 based on the PSMA-binding entity and a DOTA-chelator. This precursor was employed for the synthesis of all ibuprofen-derivatized PSMA ligands.

**Synthesis of precursor 5** (Scheme 1): The urea-based PSMA-binding entity and the linker moiety were synthesized as previously reported 14. A joint precursor (**5**) was synthesized for all four PSMA ligands. For this purpose, resin-immobilized compound **1** (0.40 mmol) was swelled in anhydrous dichloromethane (DCM) for 45 min and conditioned in *N,N*-dimethylformamide (DMF). Subsequently, 4.0 equiv *N_ɑ_-*Fmoc-N_ε_-Alloc-L-lysine (Fmoc-Lys(Alloc)-OH, 1.6 mmol) were activated with 3.96 equiv *O*-(benzotriazol-1-yl)-*N,N,N',N'*-tetramethyluronium hexafluorophosphate (HBTU, 1.58 mmol) in the presence of 4.0 equiv *N,N*-diisopropylethylamine (DIPEA, 1.6 mmol) in anhydrous DMF. Two minutes after the addition of DIPEA, the activated solution was added to compound **1** and agitated for 1 h to give resin-immobilized compound **2**. The selective removal of the *N_α_*-Fmoc-protecting group of the lysine residue was performed with a mixture of DMF and piperidine in a ratio of 1:1 (*v/v*) twice for 5 min. The resulting compound **3** on the resin was washed with DMF. Subsequently, 3.0 equiv 2-(4,7,10-tris(2-(*t*-butoxy)-2-oxoethyl)-1,4,7,10-tetraazacyclo-dodecan-1-yl)acetic acid (DOTA-tris(*t*Bu)ester; 1.2 mmol) were activated with 2.97 equiv HBTU (1.19 mmol) in the presence of 4.0 equiv DIPEA (1.6 mmol) in anhydrous DMF for two minutes. The activated solution was added to resin-immobilized compound **3** and the reaction mixture was agitated for 2-4 h. The resulting compound **4** was washed with DMF and cleavage of the *N_ε_*-Alloc protecting group was performed with 0.03 equiv tetrakis(triphenylphosphine)palladium(0) (Pd(PPh_3_)_4_, 0.01 mmol) in the presence of 30 equiv morpholine (12 mmol) in DCM within 2 h in the dark. To remove residuals of palladium, the resin-immobilized compound was additionally washed with 1% DIPEA in DMF and then with a solution of sodium diethyldithiocarbamate (15 mg/mL) in DMF. Precursor **5** was split equally into four portions for conjugation of (*RS*)*-*ibuprofen directly or via an amino acid residue on the *N_ɛ_*-group of the lysine side chain.

**Scheme 2:**
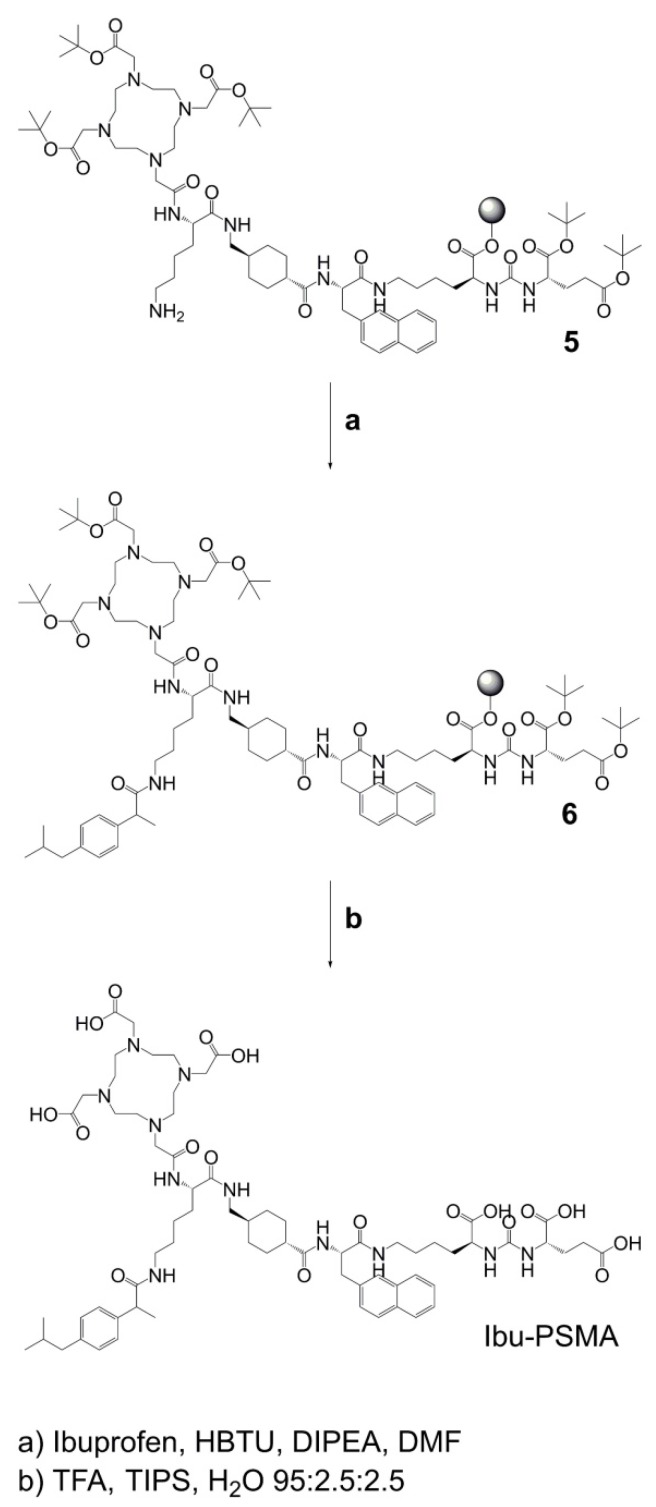
Synthesis of Ibu-PSMA based on resin-immobilized precursor 5.

**Synthesis of Ibu-PSMA** (Scheme 2): Resin-immobilized precursor **5** (0.10 mmol) was swelled in anhydrous DCM for 45 min and conditioned in DMF. Then, 6.0 equiv of a racemic mixture of 2-(4-(2-methylpropyl)phenyl)propanoic acid ((*RS*)-ibuprofen, 0.60 mmol) were activated with 5.94 equiv HBTU (0.59 mmol) in the presence of 6.0 equiv DIPEA (0.60 mmol) in anhydrous DMF. After two minutes activation time, the solution was added to precursor **5** and agitated for 2 h. The resulting resin-immobilized compound **6** was washed with DMF, DCM, and diethyl ether (Et_2_O) and dried under vacuum. The product was cleaved from the resin and simultaneously deprotected with a mixture consisting of trifluoroacetic acid (TFA), triisopropylsilane (TIPS) and Milli-Q water in a ratio of 95:2.5:2.5 (*v/v*) within 3-6 h. TFA was evaporated, the crude compound dissolved in acetonitrile (ACN) and Milli-Q water in a ratio of 1:2 (*v/v*) and purified by HPLC to yield Ibu-PSMA (Supplementary Material).

**Scheme 3:**
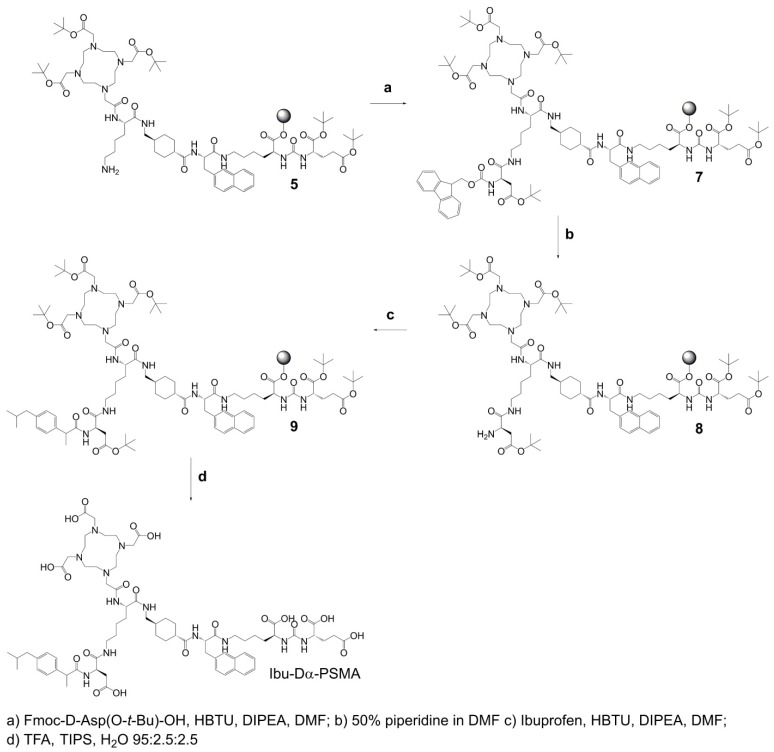
Synthesis of Ibu-Dα-PSMA based on resin-immobilized precursor 5.

**Synthesis of Ibu-Dα-PSMA** (Scheme 3): For the synthesis of Ibu-Dα-PSMA, resin-immobilized precursor **5** (0.10 mmol) was swelled in anhydrous DCM for 45 min and conditioned in DMF. Relative to precursor **5**, 4.0 equiv Fmoc-D-aspartic acid 4-*tert*-butyl ester (Fmoc-D-Asp(O-*t*-Bu)-OH, 0.40 mmol) were activated using 3.96 equiv HBTU (0.40 mmol) in the presence of 4.0 equiv DIPEA (0.40 mmol) in anhydrous DMF. Two minutes after addition of DIPEA, the activated solution was given to the resin and agitated for 2 h to give resin-immobilized compound **7**. It was washed with DMF and the *N_α_*-Fmoc-protecting group cleaved by agitating with a mixture of DMF and piperidine in a ratio of 1:1 (*v*/*v*) twice for 5 min to yield compound **8**. The resin-immobilized compound was washed again with DMF. (*RS*)-Ibuprofen (4.0-6.0 equiv; 0.40-0.60 mmol) was activated using 3.96-5.94 equiv HBTU (0.40-0.59 mmol) in the presence of 4.0-6.0 equiv DIPEA (0.40-0.60 mmol) in anhydrous DMF. After two minutes activation time, the solution was added to resin-immobilized compound **8** and agitated up to 2 h. Subsequently, the resin was washed with DMF, DCM and Et_2_O, respectively, and dried under reduced pressure. Compound **9** was cleaved from the resin and simultaneously deprotected with a mixture consisting of TFA, TIPS and Milli-Q water in a ratio of 95:2.5:2.5 (*v*/*v*) within 3-6 h. TFA was evaporated, the crude compound dissolved in ACN and Milli-Q water in a ratio of 1:2 (*v*/*v*) and purified by RP-HPLC to yield Ibu-Dα-PSMA (Supplementary Material).

**Synthesis of Ibu-N-PSMA and Ibu-DAB-PSMA:** The syntheses and purification of Ibu-N-PSMA and Ibu-DAB-PSMA were performed in analogy to the synthesis of Ibu-Dα-PSMA while using different linker entities (Supplementary Material, Schemes S1 and S2). For the synthesis of Ibu-N-PSMA, Fmoc-N-trityl-D-asparagine (Fmoc-D-Asn(Trt)-OH, 0.40 mmol) was used instead of Fmoc-D-Asp(O-*t*-Bu)-OH in the first coupling step. Ibu-DAB-PSMA was synthesized by using *N_ɑ_-*Fmoc-N_ε_-boc-D-diaminobutyric acid (DAB, Fmoc-D-Dab(Boc)-OH, 0.40 mmol) instead of Fmoc-D-Asp(O-*t*-Bu)-OH.

### Radiolabeling and radioligand identification

The radiolabeling of the novel PSMA ligands (Ibu-PSMA, Ibu-Dα-PSMA, Ibu-N-PSMA and Ibu-DAB-PSMA) with ^177^Lu (no-carrier added ^177^Lu in 0.05 M HCl; Isotope Technologies Garching ITG GmbH, Germany) was performed at pH 4.5 in a 1:5 (*v/v*) mixture of sodium acetate (0.5 M, pH 8) and HCl (0.05 M, pH ~1). The reaction mixture was incubated for 10 min at 95 °C, followed by a quality control using HPLC for the determination of the radiochemical purity of the radioligands as previously reported (Supplementary Material) 23, 29. For the *in vitro* and *in vivo* studies, the radioligands were prepared at molar activities of 5‒50 MBq/nmol depending on the experiment. In order to investigate the option of radioligand preparation at higher molar activity, each ligand was labeled at 100 MBq/nmol for at least three times in order to determine the resulting radiochemical purity.

For identification of the radiolabeled ligands, non-radioactive reference compounds were prepared by labeling the PSMA ligands with ^175^Lu. The formation of ^175^Lu-PSMA ligands was assessed by analyzing the reaction mixture using HPLC with a UV detector and by LC-MS in order to identify the correct mass (Supplementary Material).

### Radiolytic stability

The radiolytic stability of the PSMA radioligands (50 MBq/nmol) was determined at a high activity concentration (250 MBq/500 μL saline) over a period of 24 h in the absence or presence of L-ascorbic acid (3 mg). The samples were kept at room temperature and aliquots were taken 1 h, 4 h and 24 h after labeling in order to determine the amount of intact radioligand using HPLC as previously reported (Supplementary Material) 23, 29.

### *n*-Octanol/PBS distribution coefficient

The *n*-octanol/PBS distribution coefficient of ^177^Lu-Ibu-PSMA, ^177^Lu-Ibu-Dα-PSMA, ^177^Lu-Ibu-N-PSMA and ^177^Lu-Ibu-DAB-PSMA in a *n*-octanol/PBS system was determined as previously reported (Supplementary Material) 23.

### Binding to mouse and human plasma proteins

Plasma protein-binding properties of ^177^Lu-Ibu-PSMA, ^177^Lu-Ibu-Dα-PSMA, ^177^Lu-Ibu-N-PSMA and ^177^Lu-Ibu-DAB-PSMA as well as ^177^Lu-PSMA-617 and ^177^Lu-PSMA-ALB-56 were investigated using an ultrafiltration assay as previously reported 23. In short, the PSMA ligands were labeled with ^177^Lu at a molar activity of 50 MBq/nmol, diluted in PBS (pH 7.4) to an activity concentration of 10 MBq/500 μL and incubated in mouse or human plasma at 37 °C for 30 min. After incubation, each sample was loaded on a centifree ultrafiltration device (4104 centrifugal filter units; Millipore, 30000 Da nominal molecular weight limit, methylcellulose micropartition membranes) and centrifuged at 2000 rpm for 40 min at 20 °C to allow the separation of the unbound and plasma-bound radioligand fractions. Samples from the loading solution and samples from the filtrate were taken and counted for radioactivity in a γ-counter. The amount of plasma-bound radioligand was calculated as the fraction of radioactivity measured in the filtrate relative to the corresponding loading solution (set to 100%). Control experiments were performed with each radioligand by incubation in PBS (pH 7.4) prior to filtration in order to determine the unspecific binding of the radioligands to the filter membrane. The experiments were performed at least three times for each radioligand. A one-way ANOVA with a Tukeys's multiple comparison post-test was used for statistical analysis in GraphPad Prism software (version 7). A p-value of <0.05 was considered statistically significant.

### Cell culture and internalization experiments

Sublines of the androgen-independent PC-3 human prostate cancer cell line, PSMA-positive PC-3 PIP and PSMA-negative PC-3 flu cells, were kindly provided by Prof. Dr. Martin Pomper (Johns Hopkins University School of Medicine, Baltimore, MD, U.S.A.) (Supplementary Material). The cells were cultured in RPMI-1640 cell culture medium supplemented with 10% fetal calf serum, L-glutamine and antibiotics. Puromycin (2 µg/mL) was added to the cell cultures to maintain PSMA expression as previously reported 32. Uptake and internalization studies of the ^177^Lu-labeled PSMA ligands were performed as previously reported (Supplementary Material) 23. Each radioligand was investigated in three independent experiments performed in triplicate with PC-3 PIP tumor cells and, in one experiment, performed in triplicate using PC-3 flu tumor cells.

### Determination of K_D_ values

The K_D_ values, indicating the PSMA-binding affinity of the radioligands, were determined using PC-3 PIP tumor cells. The cells were seeded in 48-well-plates (8 x 10^4^ cells in 500 µL RPMI medium/well) allowing adhesion and growth overnight. During the experiment, well plates were kept on ice. After removal of the supernatant, the cells were washed once with ice-cold PBS (pH 7.4) before addition of different concentrations (1‒2000 nM) of the radiolabeled PSMA ligands (5 MBq/nmol) in ice-cold RPMI medium without supplements. Some of the PC-3 PIP tumor cell samples were co-incubated with 2-phosphonomethyl pentanedioic acid (2-PMPA, 200 μM) to block PSMA enabling the determination of non-specific binding. After incubation of the well-plates for 30 min at 4 °C, the supernatants were removed and the cells washed twice with ice-cold PBS. The cells were lysed (NaOH, 1 M, 600 µL) and transferred to 4 mL tubes for counting the activity in a γ-counter (Wallac Wizard 1480, Perkin Elmer). The K_D_ values were determined by plotting specific binding (total binding minus unspecific binding determined with blocking agent) against the molar concentration of the added radioligands. The nonlinear regression analysis was performed using GraphPad Prism software (version 7). Statistical significance was assessed using a one-way ANOVA with a Tukeys's multiple comparison post-test in GraphPad Prism software (version 7). A p-value of <0.05 was considered statistically significant.

### *In vivo* studies

All applicable international, national, and/or institutional guidelines for the care and use of animals were followed. In particular, all animal experiments were carried out according to the guidelines of Swiss Regulations for Animal Welfare. The preclinical studies have been ethically approved by the Cantonal Committee of Animal Experimentation and permitted by the responsible cantonal authorities.

Female athymic nude BALB/c mice were obtained from Charles River Laboratories, Sulzfeld, Germany, at the age of 5-6 weeks. Since PC-3 PIP/flu tumor cells are androgen-independent, female mice were used, which is the commonly preferred gender for animal experiments as they are housed more peacefully in groups than male mice. The mice were subcutaneously inoculated with PSMA-positive PC-3 PIP cells (6 x 10^6^ cells in 100 μL Hank's balanced salt solution (HBSS)) on the right shoulder and with PSMA-negative PC-3 flu cells (5 x 10^6^ cells in 100 μL HBSS) on the left shoulder as previously reported 23, 29. Two weeks later, the tumors reached a size of about 100-300 mm^3^ suitable for biodistribution and imaging studies.

### Biodistribution studies

The PSMA ligands were labeled at a molar activity of 5 MBq/nmol and diluted in saline containing 0.05% bovine serum albumin (BSA) in order to prevent adherence of the radioligands to vials and syringes. Tumor-bearing mice were intravenously injected with the respective radioligand (5 MBq, 1 nmol, 100 µL). Groups of 3‒6 mice were sacrificed at 1 h, 4 h, 24 h, 48 h, 96 h or 192 h p.i. Selected organs and tissues were collected, weighed and measured using a γ-counter (Perkin Elmer, Wallac Wizard 1480). The results were decay-corrected and listed as percentage of the injected activity per gram of tissue mass (% IA/g). Data presented as the average ± standard deviation (SD). The area under the curve (AUC) was determined for the uptake of all four radioligands in PC-3 PIP tumors, kidneys, liver, blood and salivary glands based on non-decay-corrected data obtained from the biodistribution experiments using GraphPad Prism software (version 7). Statistical significance was assessed using a one-way ANOVA with a Tukeys's multiple comparison post-test in GraphPad Prism software (version 7). A p-value of <0.05 was considered statistically significant.

### SPECT/CT imaging studies

SPECT/CT experiments were performed using a dedicated small-animal SPECT/CT camera (NanoSPECT/CT^TM^, Mediso Medical Imaging Systems, Budapest, Hungary) as previously reported (Supplementary Material) 23, 32. The PSMA ligands were labeled at a molar activity of 25 MBq/nmol and diluted in saline containing 0.05% BSA. Scans were acquired 4 h and 24 h after injection of the radioligands (25 MBq, 1 nmol, 100 µL). Data were reconstructed using NanoSPECT/CT^TM^ software and post-processed using VivoQuant (version 3.0, inviCRO Imaging Services and Software, Boston USA). A Gauss post-reconstruction filter (FWHM = 1 mm) was applied and the scale of radioactivity was set as indicated on the images (minimum value = 0.7 Bq/voxel to maximum value = 70 Bq/voxel).

## Results

### Synthesis of the PSMA ligands

The PSMA ligands with an albumin-binding moiety were synthesized via a solid-phase platform according to the method previously reported for the synthesis of other PSMA ligands 29. A multistep synthesis (17 steps for Ibu-PSMA and 19 steps Ibu-Dα-PSMA, Ibu-N-PSMA and Ibu-DAB-PSMA, respectively) provided these ligands in isolated overall yields of 3-15% after HPLC purification. The PSMA ligands were characterized by analytical HPLC and MALDI-TOF-MS (Bruker UltraFlex II). The chemical purity of the ligands was >99% (Table 1).

### Radiolabeling of PSMA ligands and identification of the radioligands

Radiolabeling of the ibuprofen-derivatized PSMA ligands with ^177^Lu at 5-50 MBq/nmol resulted in high radiochemical purity (≥96% for ^177^Lu-Ibu-PSMA and ≥99% for ^177^Lu-Ibu-Dα-PSMA, ^177^Lu-Ibu-N-PSMA and ^177^Lu-Ibu-DAB-PSMA) of the radioligands, which were used for *in vitro* and *in vivo* studies without further purification steps (Supplementary Material, Figure S1). The results of the radiolabeling experiments performed at a molar activity of 100 MBq/nmol revealed high radiochemical purity of 96 ± 2% for ^177^Lu-Ibu-PSMA, 98 ± 1% for ^177^Lu-Ibu-Dα-PSMA, 97 ± 1% for ^177^Lu-Ibu-N-PSMA and 96 ± 4% for ^177^Lu-Ibu-DAB-PSMA.

The identity of ^175^Lu-PSMA ligands was confirmed using LC-MS. The retention times of the single ^175^Lu-PSMA ligands using HPLC with a UV-detector corresponded well with the retention times of the radioactive analogues (Supplementary Material, Figure S2 and Table S1).

### Determination of the stability and *n*-octanol/PBS distribution coefficients

At high activity concentration, similar to how it would be used clinically; radiolytic degradation was observed for all ibuprofen-derivatized radioligands after 24 h when incubated in saline only. The HPLC chromatograms performed for quality control showed multiple degradation products of unknown structure and only ≤20% intact radioligand. The addition of L-ascorbic acid, which is commonly added to formulations of radioligands for patients' application, was able to almost entirely prevent radiolytic processes resulting in ≥91% intact radioligands after 24 h incubation time (Supplementary Material, Figure S3).

All radioligands were characterized with hydrophilic properties resulting in logD values of -2.2 ± 0.1, -2.2 ± 0.1 and -2.4 ± 0.1 for ^177^Lu-Ibu-PSMA, ^177^Lu-Ibu-N-PSMA and ^177^Lu-Ibu-DAB-PSMA, respectively. ^177^Lu-Ibu-Dα-PSMA was slightly more hydrophilic as demonstrated by the logD value of -3.0 ± 0.3. The logD values of the ibuprofen-based PSMA ligands were all in the same range as the logD value determined for ^177^Lu-PSMA-ALB-56 (-2.9 ± 0.2) 29, but higher than the value determined for ^177^Lu-PSMA-617 (-4.4 ± 0.2) 23.

### Binding properties to mouse and human plasma proteins

**Figure 2:**
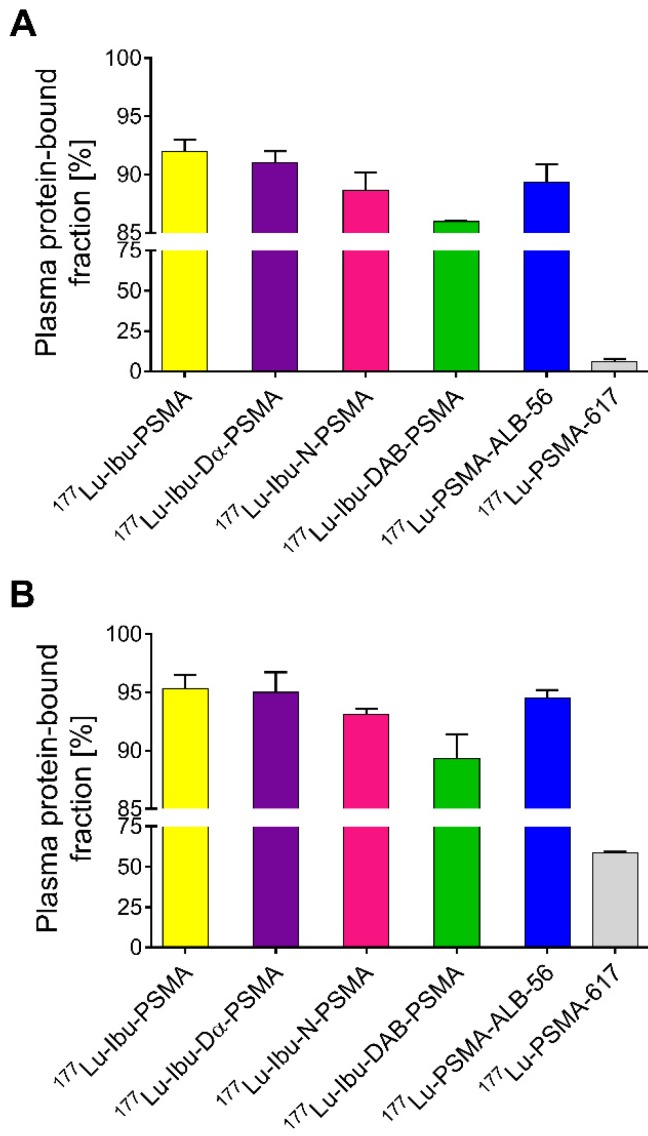
** Plasma protein-binding properties of the ibuprofen-derivatized radioligands, ^177^Lu-PSMA-ALB-56 and ^177^Lu-PSMA-617.** The values show the average ± SD of three independent experiments. **(A)** Binding properties of radioligands to mouse plasma proteins; **(B)** Binding properties of radioligands to human plasma proteins.

Ultrafiltration experiments were performed to determine the plasma protein-binding properties of the ibuprofen-derivatized PSMA radioligands in comparison to ^177^Lu-PSMA-ALB-56 and ^177^Lu-PSMA-617, respectively (Figure 2) 23, 29. The binding to mouse plasma proteins of the ibuprofen-derivatized radioligands revealed values between 86-92%, which were in the same range as the binding of ^177^Lu-PSMA-ALB-56 (89 ± 2%; p>0.05). It was observed that ^177^Lu-Ibu-DAB-PSMA bound significantly less to mouse plasma than ^177^Lu-Ibu-PSMA (p<0.05) and ^177^Lu-Ibu-Dα-PSMA (p<0.05) and slightly less than ^177^Lu-Ibu-N-PSMA (p>0.05). All radioligands revealed a significantly increased binding to mouse plasma proteins when compared to ^177^Lu-PSMA-617, which showed a mouse protein-bound fraction of only 6.0 ± 2% (p<0.05) (Figure 2A).

^177^Lu-Ibu-PSMA, ^177^Lu-Ibu-Dα-PSMA and ^177^Lu-Ibu-N-PSMA demonstrated similar binding to human plasma proteins (93-95%) as ^177^Lu-PSMA-ALB-56 (95 ± 1%; p>0.05). ^177^Lu-Ibu-DAB-PSMA was the only radioligand that showed reduced binding to human plasma proteins (89 ± 2%; p<0.05) when compared to ^177^Lu-PSMA-ALB-56 and to the other ibuprofen-based radioligands. ^177^Lu-PSMA-617 showed a significantly lower binding to human plasma proteins (59 ± 1%; p<0.05) than all other radioligands, however, the bound fraction of ^177^Lu-PSMA-617 was clearly higher in human plasma than in mouse plasma as already previously reported 23, 29 (Figure 2B).

In control experiments performed with radioligands incubated in PBS, it was demonstrated that the fraction of free radioligand (i.e. radioligands not bound to plasma proteins) was ≤2% in the case of ^177^Lu-Ibu-PSMA, ^177^Lu-Ibu-Dα-PSMA and ^177^Lu-PSMA-617. In the case of ^177^Lu-Ibu-N-PSMA and ^177^Lu-Ibu-DAB-PSMA, the unspecific binding to the filter membrane was ~4% and ~8%, respectively, which may have led to a slight overestimation (≤8%) of the reported bound fraction (Supplementary Material, Table S2).

### Cell uptake, internalization and PSMA-affinity

The uptake of all radioligands into PC-3 PIP tumor cells was comparable to ^177^Lu-PSMA-617 after incubation for 2 h or 4 h, respectively (Supplementary Material, Figure S4A). The internalized fraction of ^177^Lu-Ibu-PSMA was slightly higher than for ^177^Lu-Ibu-Dα-PSMA, ^177^Lu-Ibu-N-PSMA, ^177^Lu-Ibu-DAB-PSMA and ^177^Lu-PSMA-617, which were all in the same range. The uptake of all radioligands in PC-3 flu tumor cells was <2% after 4 h, which indicated PSMA-specific cell uptake in PC-3 PIP cells (Supplementary Material, Figure S4B). Control experiments (performed with two of the radioligands) without addition of BSA revealed that the uptake and internalization of the radioligands were not influenced by the presence of 0.00125% BSA in the incubation solution (Supplementary Material, Figure S5). It was also demonstrated that higher concentrations of BSA or the use of HSA did not influence the uptake and the internalization of the radioligands (Supplementary Material, Figures S6).

The K_D_ values of the ibuprofen-derivatized PSMA radioligands were in the same range (p>0.05) and also not significantly different from the K_D_ value of ^177^Lu-PSMA-ALB-56 (Table 2). The K_D_ values of the albumin-binding PSMA radioligands were consistently higher than the K_D_ value of ^177^Lu-PSMA-617 determined under the same experimental conditions (Table 2). According to the applied statistical analysis, the difference was, however, not significant (p>0.05).

### Biodistribution study

The tissue distribution profiles of ^177^Lu-Ibu-PSMA, ^177^Lu-Ibu-Dα-PSMA, ^177^Lu-Ibu-N-PSMA and ^177^Lu-Ibu-DAB-PSMA were investigated in PC-3 PIP/flu tumor-bearing mice over a period of 8 days (192 h p.i.) (Supplementary Material, Tables S3-S6, Figure S7). The data of the most important organs and tissues over the first 48 hours are listed in Table 3. Accumulation of activity in PC-3 PIP tumors was fast and reached a maximum accumulation of 65-81% IA/g between 4 h and 24 h after injection of the radioligands. Activity was retained in the tumor over the course of the study resulting in values >15% IA/g at eight days post injection in all cases. Clearance of activity from tumor tissue was slowest in the case of ^177^Lu-Ibu-DAB-PSMA, which resulted in the highest tumor uptake of the ibuprofen-containing radioligands eight days after injection. The uptake in PSMA-negative PC-3 flu tumors was low (<10% of the uptake in the PC-3 PIP tumor at 1 h p.i.) and decreased to background levels over time, which confirmed that the uptake in PC-3 PIP tumors was PSMA-specific.

One hour after injection, the radioligands showed blood activity levels of 13-18% IA/g. Efficient clearance of activity from the blood pool resulted in ≤6% IA/g at 4 h and <0.6% at 24 h after injection of the radioligands. Kidney uptake was lowest for ^177^Lu-Ibu-DAB-PSMA at all measured time points with an accumulated activity of 19 ± 2% and 6.0 ± 0.7% IA/g at 4 h and 24 h p.i, respectively. ^177^Lu-Ibu-N-PSMA also showed relatively low values of 27 ± 8% and 8.0 ± 1% IA/g at 4 h and 24 h p.i., respectively. Both radioligands were efficiently cleared from the kidneys, resulting in renal retention below 2.5% IA/g after 96 h. The other radioligands showed a higher kidney retention and/or slower clearance. Accumulation of activity in the salivary glands was generally low (3.2-4.6% IA/g at 1 h p.i.) and cleared rapidly over time (0.09-0.42% IA/g at 48 h p.i.). Radioactivity levels in all other tissues were low, mostly below blood levels and decreased over time (Supplementary Material, Figure S7).

Control experiments using ^177^Lu-Ibu-PSMA without addition of BSA to the injection solution revealed that the tissue distribution of the radioligand was not substantially influenced by the presence of 0.05% BSA in the injection solution (Supplementary Material, Table S7). Addition of BSA to the injection solution is, however, of relevance to prevent adherence of the radioligands to vials and syringes and, hence, ensure the injection of accurate quantities.

**Figure 3:**
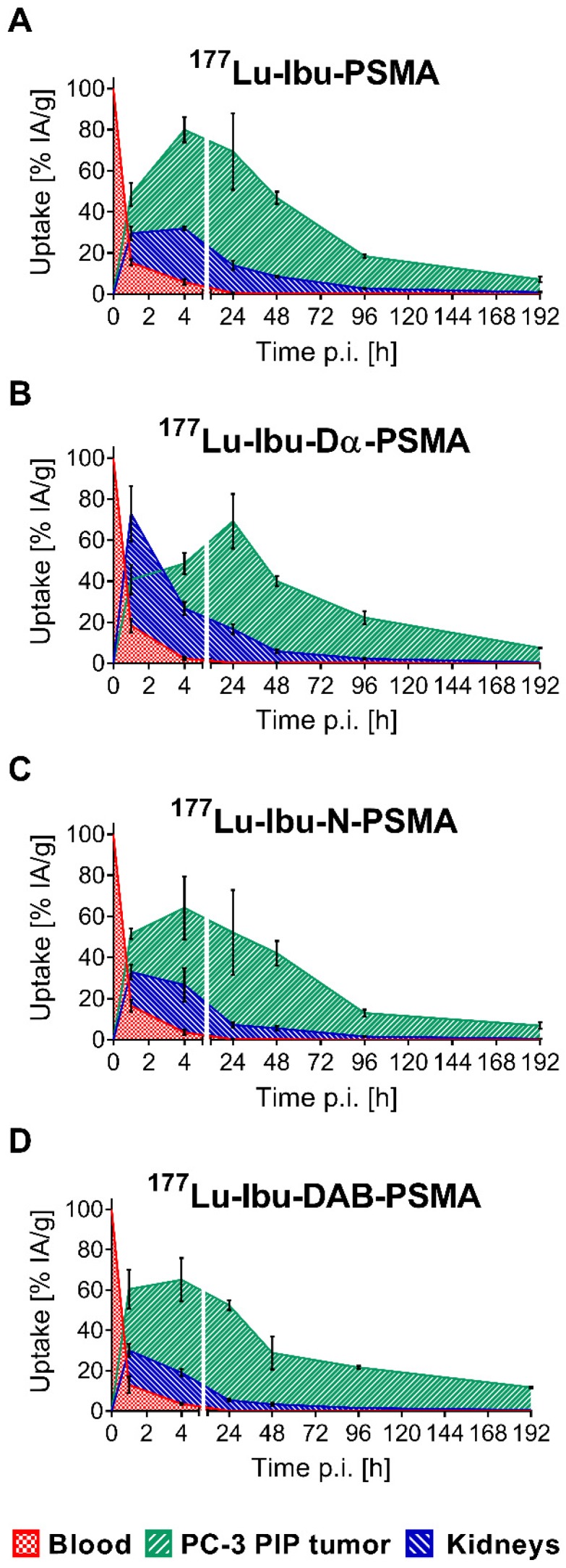
** Area under the curves (AUCs) of the ibuprofen-derivatized radioligands.** AUCs were calculated based on non-decay-corrected biodistribution data up to 192 h p.i. (8 d p.i.) of all novel radioligands. **(A)** ^177^Lu-Ibu-PSMA, **(B)** ^177^Lu-Ibu-Dα-PSMA, **(C)** ^177^Lu-Ibu-N-PSMA, **(D)** ^177^Lu-Ibu-DAB-PSMA. Each data point represents the average of a group of mice ± SD (n = 3-6) indicated as percentage of injected activity per gram tissue [% IA/g].

**Figure 4:**
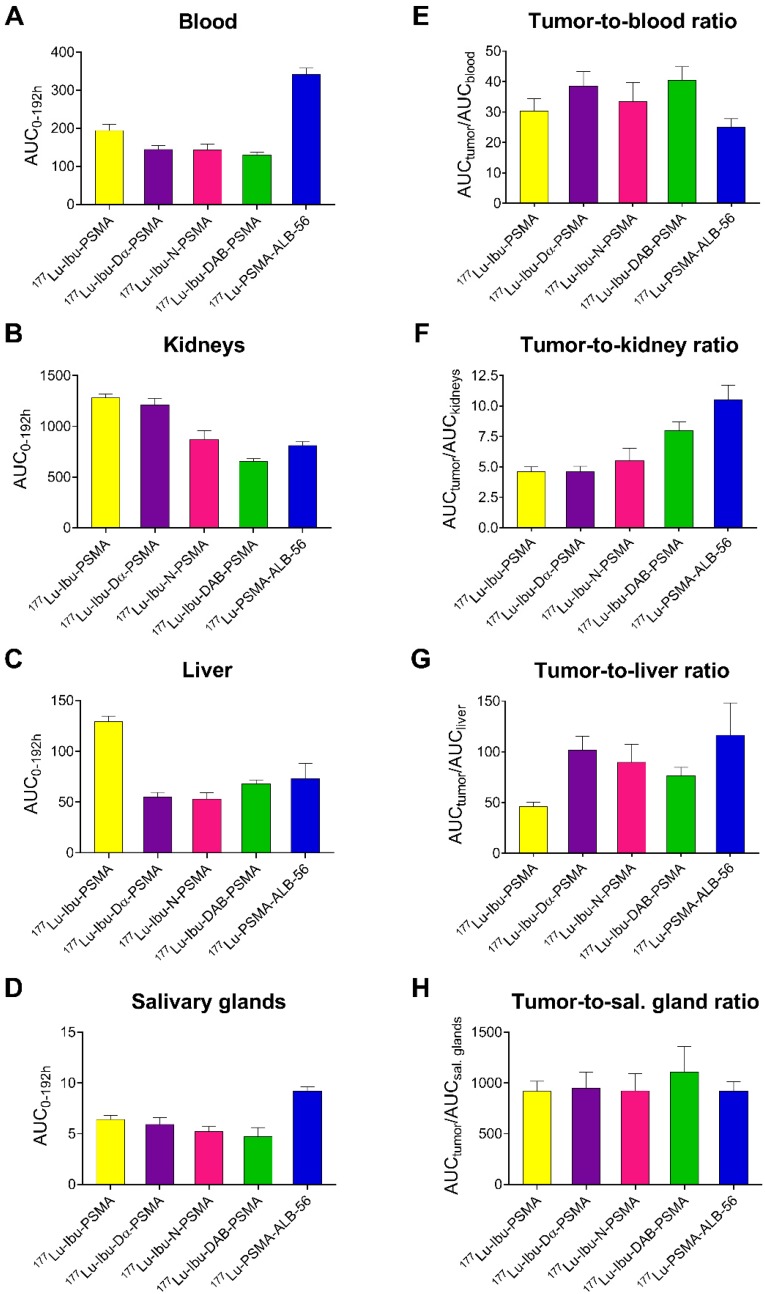
AUC values [(% IA/g)*h] and AUC ratios of selected organs and tissues. (A) AUC of activity retention in blood, **(B)** AUC of activity retention in the kidneys, **(C)** AUC of activity retention in the liver and **(D)** AUC of activity retention in the salivary glands; (**E**) tumor-to-blood AUC ratios, **(F)** tumor-to-kidney AUC ratios, **(G)** tumor-to-liver AUC ratios and (**H**) tumor-to-salivary gland ratios of AUC values obtained for each radioligand.

Areas under the curve (AUCs) were determined based on non-decay-corrected biodistribution data for selected organs and tissues enabling the comparison of the distribution properties of the radioligands (Figure 3; Supplementary Material, Table S8). The ratios of the AUC values for the tumor relative to the blood, kidneys, liver and salivary glands, respectively, were calculated as an indirect measure for the absorbed dose ratios (Figure 4; Supplementary Material, Table S8).

The AUC values representing the PC-3 PIP tumor uptake after injection of the ibuprofen-derivatized radioligands revealed similar values (p>0.05), with the only exception of ^177^Lu-Ibu-N-PSMA, which showed a significantly lower AUC value for the tumor uptake (p<0.05). The 1.3- to 1.6-fold increased AUC values for the PC-3 PIP tumor uptake of the ibuprofen-derivatized radioligands were significantly higher as compared to the previously reported tumor uptake of ^177^Lu-PSMA-617 (p<0.05) 23. Importantly, the lowest blood activity was determined for ^177^Lu-Ibu-DAB-PSMA, which resulted in up to 1.3-fold increased tumor-to-blood AUC ratios compared to the other ibuprofen-derivatized radioligands (p>0.05) and a 1.6-fold increased tumor-to-blood AUC ratio compared to the previously published value for ^177^Lu-PSMA-ALB-56 (p<0.05) (Figure 4; Supplementary Material, Table S8) 29. The AUC values determined for the kidneys were lowest for ^177^Lu-Ibu-DAB-PSMA (p<0.05), resulting in a 1.5- to 1.7-fold increased tumor-to-kidney AUC ratio when compared to ^177^Lu-Ibu-N-PSMA, ^177^Lu-Ibu-Dα-PSMA and ^177^Lu-Ibu-PSMA, respectively. In comparison to the previously developed ^177^Lu-PSMA-ALB-56 29, the ibuprofen-derivatized radioligands showed slightly reduced tumor-to-kidney AUC ratios (p>0.05), however, the absolute AUC value for the kidneys was lower for ^177^Lu-Ibu-DAB-PSMA as compared to all other albumin-binding radioligands including ^177^Lu-PSMA-ALB-56. The AUC value for the salivary glands was also lowest for ^177^Lu-Ibu-DAB-PSMA, leading to the highest tumor-to-salivary gland AUC ratio of all investigated albumin-binding radioligands (Supplementary Material, Table S8).

### SPECT/CT imaging studies

**Figure 5:**
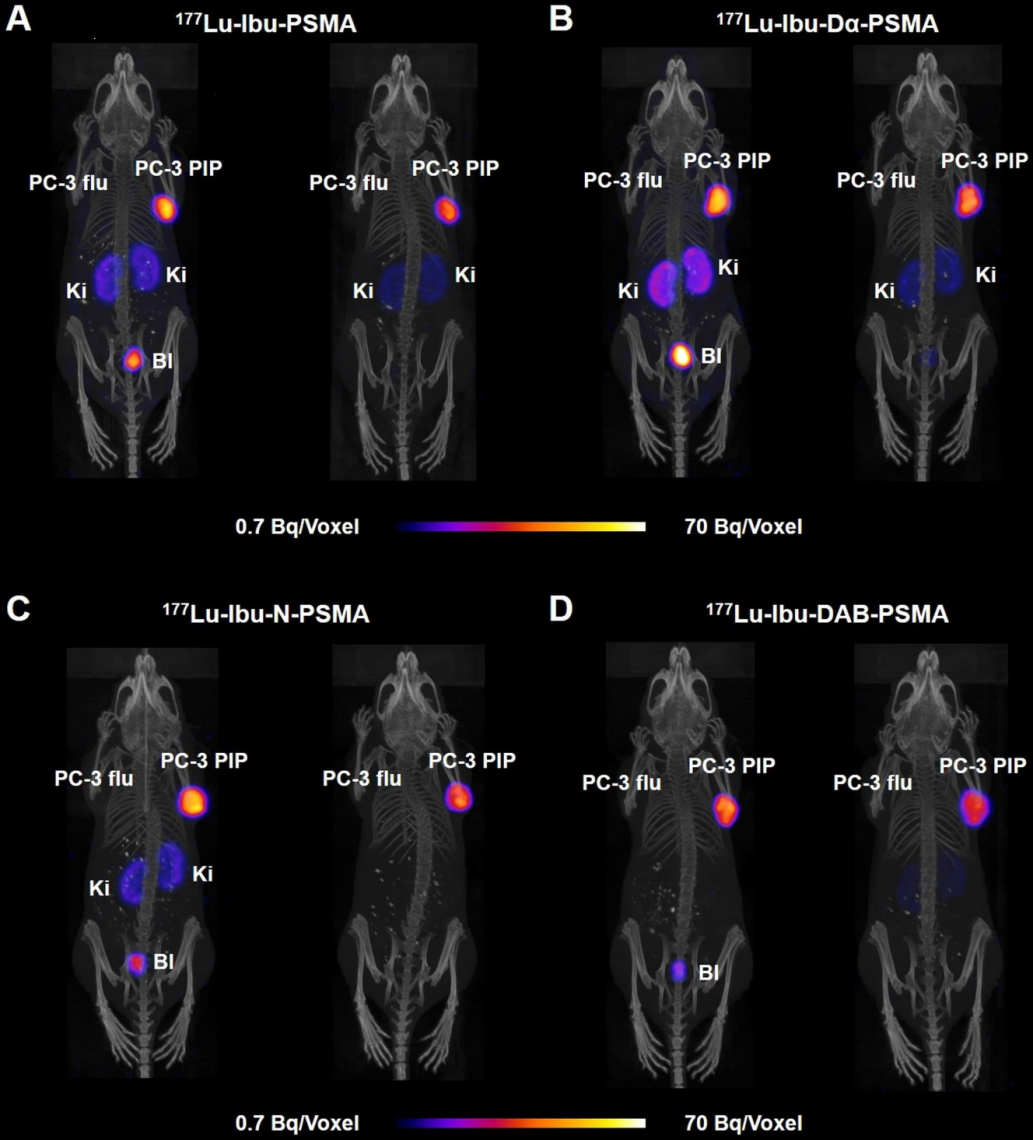
** SPECT/CT images of the ibuprofen-derivatized radioligands in PC-3 PIP/flu tumor-bearing mice.** Images are shown as maximum intensity projections (MIPs) at 4 h (left) and 24 h (right) after injection of ^177^Lu-labeled PSMA ligands (labeled at 25 MBq/nmol). **(A)** ^177^Lu-Ibu-PSMA (21 MBq injected); **(B)** ^177^Lu-Ibu-Dα-PSMA (23 MBq injected); **(C)** ^177^Lu-Ibu-N-PSMA (24 MBq injected) and **(D)** ^177^Lu-Ibu-DAB-PSMA (25 MBq injected); PC-3 PIP = PSMA-positive tumor; PC-3 flu = PC-negative tumor; Ki = kidney; Bl = urinary bladder.

SPECT/CT images of PC-3 PIP/flu tumor-bearing mice were performed 4 h and 24 h after injection of the ^177^Lu-labeled PSMA ligands (~25 MBq; Figure 5). Accumulation of all radioligands in PC-3 PIP xenografts was similar at 4 h p.i., however, renal uptake was lowest in the case of ^177^Lu-Ibu-DAB-PSMA. On the other hand, ^177^Lu-Ibu-Dα-PSMA showed the highest retention in the kidneys, both at 4 h and 24 h p.i. The excretion profile of ^177^Lu-Ibu-N-PSMA and ^177^Lu-Ibu-DAB-PSMA was faster as compared to the excretion of ^177^Lu-Ibu-PSMA and ^177^Lu-Ibu-Dα-PSMA. In line with the biodistribution data, no activity accumulation was detected in PSMA-negative PC-3 flu tumors.

## Discussion

In this study, a new class of PSMA radioligands with albumin-binding properties was developed with the aim to achieve high tumor uptake, but reduce the accumulation of radioactivity in the blood pool to the lowest possible level. The structure of the anti-inflammatory drug ibuprofen was used as an albumin-binding entity. Importantly, any pharmacologic activity of ibuprofen after conjugation to the PSMA ligands can be excluded, not only because of the low quantities that would be applied, but also because of the fact that the (no longer available) carboxyl group would be essential for its binding to the cyclooxygenases (COX) 33, 34.

Ibuprofen was directly conjugated to the PSMA ligand (Ibu-PSMA) or by using negatively (Ibu-Dα-PSMA), neutral (Ibu-N-PSMA) or positively charged amino acid-based linker entities (Ibu-DAB-PSMA). In order to exclude any influence of the linker size on the characteristics of the radioligands, we chose to use amino acids with equal side chain length; hence, instead of using lysine, the unnatural amino acid diaminobutyric acid (DAB) was employed.

All four ligands were prepared by solid-phase support technique as previously reported for other PSMA ligands 23, 29, enabling fast and convenient synthesis while avoiding multiple purifications after each conjugation step. The only HPLC-based purification step was performed with the final compounds to obtain highly pure products that could be labeled with ^177^Lu at molar activities up to 100 MBq/nmol. Radiolytic degradation at high activity concentrations was more pronounced than it was previously observed with albumin-binding PSMA radioligands 23, 29. This indicated that the ibuprofen did not share the stabilizing effect provided by the *p*-iodophenyl or *p*-tolyl entity. Addition of L-ascorbic acid to the incubation solution, as it is commonly used in formulations for patients, ensured, however, maintaining over 91% intact radioligand over 24 hours as it was also the case for ^177^Lu-PSMA-617. PSMA-binding affinity defined by determination of K_D_ values was in the same range for all investigated radioligands, with the highest affinity observed for ^177^Lu-PSMA-617. The slightly reduced affinity determined for PSMA radioligands with an albumin-binding entity may be ascribed to the common characteristic that they adhere to the material of well-plates and tubes, which may have affected the accuracy of the assay.

Ultrafiltration experiments performed with mouse and human plasma demonstrated pronounced protein-binding properties of three of the ibuprofen-derivatized radioligands similar to the previously published results obtained with ^177^Lu-PSMA-ALB-56 29. ^177^Lu-Ibu-DAB-PSMA showed slightly reduced binding to serum albumin. This may be explained by potential repulsive forces between the positively charged linker of ^177^Lu-Ibu-DAB-PSMA and the positively charged amino acid side chains present in the binding pocket of albumin 35. Consistent with the findings previously reported for ^177^Lu-PSMA-ALB-56 29, the binding of the ibuprofen-derivatized radioligands to mouse plasma proteins was only slightly lower than to human plasma proteins. This discrepancy is most likely due to different structural characteristics of mouse and human plasma proteins 36, 37. ^177^Lu-PSMA-617, which does not comprise a dedicated albumin binder, showed almost no binding to mouse plasma proteins but low binding to human plasma proteins as previously reported 23, 29. A hydrophobic interaction of 2-naphthylamine with BSA was previously reported 38, hence, it may be speculated that the naphthyl entity in the linker entity of ^177^Lu-PSMA-617 interacts with serum albumin and that this interaction differs between plasma proteins of different species.

Based on experimental data and theoretical calculations, ibuprofen preferentially binds to albumin in its charged form 39. Our findings showed, however, that despite the loss of the carboxylate function due to the conjugation to the PSMA molecule, the albumin-binding properties were still sufficient to enable the desired binding to mouse and human plasma proteins. These results are in line with those obtained for an anticoagulant peptide bearing N-terminal ibuprofen, which resulted in a significantly increased *in vivo* half-life and fraction of albumin-bound peptide 40, 41.

Biodistribution studies in mice confirmed the radioligands' potential to bind to plasma proteins as demonstrated by an enhanced blood circulation time and an increased tumor accumulation compared to ^177^Lu-PSMA-617. Additional experiments were performed to determine the excretion of radioligand from the whole body of non-tumor-bearing mice (Supplementary Material, Figure S8). It was observed that body retention was reduced for the ibuprofen-equipped radioligands as compared to ^177^Lu-PSMA-ALB-56. It is, however, noteworthy that several aspects may have an influence on the pharmacokinetic properties of a radioligand, hence, the albumin-binding properties of a radioligand should not be considered as the only predictor of its tissue distribution profile. In agreement with the performed cell experiments, accumulation of the radioligands was high in PSMA-positive PC-3 PIP tumors, but negligible in PSMA-negative PC-3 flu tumors in which the uptake was below blood levels. In spite of the slightly increased lipophilicity of the four ibuprofen-bearing radioligands compared to ^177^Lu-PSMA-617, we did not observe significant accumulation in non-targeted organs and tissues. Notably, blood activity levels in mice injected with any of the ibuprofen-derivatized PSMA radioligands were considerably lower than those observed for ^177^Lu-PSMA-ALB-56. It is, however, a fact that high tumor uptake of an albumin-binding radioligand correlate with higher accumulation in background organs, hence, tumor-to-background ratios are commonly lower for albumin-binding radioligands as compared to conventional radioligands such as ^177^Lu-PSMA-617.

The accumulated activity in salivary glands, which are among the most critical organs in PSMA-targeted radioligand therapy, was negligible for all investigated radioligands. It has to be mentioned, however, that the salivary gland uptake in mice is generally low and, therefore, not predictive for the uptake in humans.

^177^Lu-Ibu-DAB-PSMA represented a good compromise between high tumor accumulation (40% higher compared to ^177^Lu-PSMA-617), but low retention of activity in other organs and tissues. Importantly, the ^177^Lu-Ibu-DAB-PSMA revealed the fastest blood and kidney clearance amongst all investigated ibuprofen-derivatized radioligands. Based on these results, it appears that a positive charge in close proximity to the albumin binder enhances the renal clearance while a negative charge at this position leads to an increased retention of activity in the kidneys. Interestingly, these findings are in line with our previous study using ^177^Lu-PSMA radioligands equipped with a *p*-iodophenyl entity, where we found the highest renal retention in the case of ligands with a negatively charged entity 23. Due to the resulting favorable tumor-to-background ratios of ^177^Lu-Ibu-DAB-PSMA, this radioligand emerged as the most promising candidate. SPECT/CT images confirmed these findings, showing clearly reduced background activity in the blood and the kidneys in the case of ^177^Lu-Ibu-DAB-PSMA.

AUC values, determined based on non-decay-corrected biodistribution data, served for comparison of the radioligands regarding the expected absorbed dose to different tissues. ^177^Lu-Ibu-DAB-PSMA revealed the most favorable tumor-to-blood and tumor-to-kidney AUC ratios among all four radioligands. Importantly, the efficient blood clearance of ^177^Lu-Ibu-DAB-PSMA resulted in a 60% increased tumor-to-blood AUC ratio for this radioligand as compared to the value previously reported for ^177^Lu-PSMA-ALB-56 29. Since this was the most critical issue to be addressed, ^177^Lu-Ibu-DAB-PSMA was identified as a more promising radioligand, even though the absolute tumor uptakes as well as the tumor-to-kidney and tumor-to-liver AUC ratios were somewhat lower than in the case of ^177^Lu-PSMA-ALB-56.

## Conclusion

In this study, we demonstrated the utilization of ibuprofen as albumin-binding entity combined with differently charged linker structures in order to optimize the tissue distribution of PSMA radioligands. ^177^Lu-Ibu-DAB-PSMA, the radioligand equipped with a positively charged diaminobutyric acid as a linker entity, showed well-balanced plasma protein-binding properties and, as a consequence, high tumor accumulation and retention but efficient clearance from the blood pool, resulting in high tumor-to-blood AUC ratios. ^177^Lu-Ibu-DAB-PSMA was, therefore, selected as the most favorable candidate of this new class of albumin-binding PSMA radioligands. These promising results warrant further preclinical investigations in view of a potential clinical translation of ^177^Lu-Ibu-DAB-PSMA.

## Supplementary Material

Supplementary methods, schemes, results, figures and tables.

## Figures and Tables

**Table 1:** Analytical data of Ibu-PSMA, Ibu-Dα-PSMA, Ibu-N-PSMA and Ibu-DAB-PSMA.

Compound	Chemical formula	MW^a^ [g/mol]	*m/z*^b^	t_r_^c^ [min]	Chemical purity^d^ [%]
Ibu-PSMA	C_68_H_99_N_11_O_18_	1358.60	1358.72	9.9	>99
Ibu-Dα-PSMA	C_72_H_104_N_12_O_21_	1473.69	1473.75	9.7	>99
Ibu-N-PSMA	C_72_H_105_N_13_O_20_	1472.70	1472.77	9.7	>99
Ibu-DAB-PSMA	C_72_H_107_N_13_O_19_	1458.72	1458.79	8.6	>99

^a^ MW: molecular weight; ^b^ *m*/*z*-peak of the unlabeled ligand obtained by mass spectrometry detected as [M + H]^+^; ^c^ Retention time of the PSMA ligand (unlabeled) on analytical HPLC. Analytical column (150 × 4.6 mm) utilized RP SunFire C18 stationary phase with mobile phases consisting of 0.1% TFA in Milli-Q water (A) and ACN (B). For analytical runs, a linear gradient of solvent A (95-5%) in solvent B at a flow rate of 1 mL/min was used over 15 min. ^d^ Determined by analytical HPLC, λ = 254 nm.

**Table 2:** K_D_ data of the ibuprofen-derivatized PSMA radioligands.

	^177^Lu-Ibu-PSMA	^177^Lu-Ibu-Dα-PSMA	^177^Lu-Ibu-N-PSMA	^177^Lu-Ibu-DAB-PSMA	^177^Lu-PSMA-ALB-56	^177^Lu-PSMA-617
K_D_ values	24 ± 7 nM	18 ± 2 nM	33 ± 17 nM	35 ± 4 nM	30 ± 6 nM	13 ± 1 nM

* There is no significant difference among the K_D_ values of the listed radioligands (p>0.05).

**Table 3:** Biodistribution data over 48 h after injection of ibuprofen-derivatized PSMA radioligands in PC-3 PIP/flu tumor-bearing mice. The values are indicated as average ± SD obtained from each group of mice (*n* = 3-6) and listed as percentage of injected activity per gram tissue [% IA/g].

	1 h p.i.	4 h p.i.	24 h p.i.	48 h p.i.
	**^177^Lu-Ibu-PSMA**			
Blood	15 ± 2	6.0 ± 1.5	0.59 ± 0.11	0.50 ± 0.02
Kidneys	30 ± 4	33 ± 1	16 ± 3	11 ± 1
Intestines	2.4 ± 0.3	1.0 ± 0.3	0.21 ± 0.05	0.14 ± 0.01
Liver	5.9 ± 0.4	2.8 ± 0.5	0.95 ± 0.11	0.85 ± 0.06
Salivary glands	4.2 ± 0.4	1.7 ± 0.4	0.32 ± 0.07	0.26 ± 0.02
Bone	2.2 ± 0.3	1.0 ± 0.2	0.17 ± 0.05	0.13 ± 0.01
PC-3 PIP Tumor	49 ± 6	81 ± 7	77 ± 21	58 ± 4
PC-3 flu Tumor	4.4 ± 1.3	2.2 ± 0.6	0.60 ± 0.19	0.51 ± 0.15
	**^177^Lu-Ibu-Dα-PSMA**			
	**1 h p.i.**	**4 h p.i.**	**24 h p.i.**	**48 h p.i.**
Blood	18 ± 3	2.3 ± 0.8	0.33 ± 0.05	0.33 ± 0.13
Kidneys	73 ± 2	27 ± 4	18 ± 3	7.2 ± 1.2
Intestines	1.6 ± 0.4	0.49 ± 0.08	0.10 ± 0.02	0.07 ± 0.02
Liver	6.5 ± 0.1	0.84 ± 0.22	0.40 ± 0.05	0.28 ± 0.06
Salivary glands	4.6 ± 0.2	0.74 ± 0.20	0.19 ± 0.05	0.42 ± 0.55
Bone	2.0 ± 0.1	0.37 ± 0.10	0.10 ± 0.02	0.07 ± 0.01
PC-3 PIP Tumor	43 ± 5	49 ± 6	77 ± 15	49 ± 4
PC-3 flu Tumor	3.2 ± 0.4	1.0 ± 0.3	0.38 ± 0.03	0.20 ± 0.09
	**^177^Lu-Ibu-N-PSMA**			
	**1 h p.i.**	**4 h p.i.**	**24 h p.i.**	**48 h p.i.**
Blood	17 ± 3	3.6 ± 1.4	0.25 ± 0.07	0.25 ± 0.04
Kidneys	33 ± 4	27 ± 9	8.0 ± 1.1	6.8 ± 1.3
Intestines	1.9 ± 0.3	0.50 ± 0.18	0.09 ± 0.02	0.10 ± 0.04
Liver	3.6 ± 0.7	1.3 ± 0.6	0.32 ± 0.05	0.46 ± 0.05
Salivary glands	3.6 ± 0.5	0.94 ± 0.38	0.57 ± 0.35	0.10 ± 0.02
Bone	2.0 ± 0.4	0.56 ± 0.22	0.08 ± 0.02	0.07 ± 0.02
PC-3 PIP Tumor	52 ± 3	65 ± 16	58 ± 21	52 ± 8
PC-3 flu Tumor	2.9 ± 0.7	1.2 ± 0.6	0.23 ± 0.01	0.15 ± 0.03
	**^177^Lu-Ibu-DAB-PSMA**			
	**1 h p.i.**	**4 h p.i.**	**24 h p.i.**	**48 h p.i.**
Blood	13 ± 4	3.7 ± 0.5	0.16 ± 0.02	0.10 ± 0.04
Kidneys	30 ± 4	19 ± 2	6.0 ± 0.7	4.1 ± 0.9
Intestines	1.6 ± 0.5	0.71 ± 0.05	0.11 ± 0.03	0.05 ± 0.01
Liver	3.1 ± 1.0	1.5 ± 0.2	0.56 ± 0.10	0.49 ± 0.08
Salivary glands	3.2 ± 1.1	0.86 ± 0.38	0.56 ± 0.37	0.09 ± 0.03
Bone	1.6 ± 0.5	0.62 ± 0.08	0.09 ± 0.02	0.06 ± 0.01
PC-3 PIP Tumor	61 ± 10	66 ± 11	52 ± 3	36 ± 10
PC-3 flu Tumor	2.7 ± 0.7	1.2 ± 0.3	0.17 ± 0.01	0.15 ± 0.04

## Abbreviations

AUC: area under the curve
Bl: bladder
BSA: bovine serum albumin
COX: cyclooxygenase
CT: computed tomography
DAB: diaminobutyric acid
DOTA: 1,4,7,10-tetraazacyclododecane-1,4,7,10-tetraacetic acid
DOTAGA: 2-[1,4,7,10-tetraazacyclododecane-4,7,10-tris(acetic acid)]-pentanedioic acid
DTPA: diethylenetriamine pentaacetic acid
FWHM: full width at half maximum
HBSS: Hank's balanced salt solution
HPLC: high performance liquid chromatography
IA: injected activity
Ki: kidney
LC-MS: liquid chromatography-mass spectrometry
MALDI: matrix-assisted laser desorption/ionization
mCRPC: metastatic castration resistant prostate cancer
MS: mass spectrometry
MW: molecular weight
NSAID: non-steroidal anti-inflammatory drug
PBS: phosphate-buffered saline
PCa: prostate cancer
p.i.: post injection
2-PMPA: 2-(phosphonomethyl)-pentandioic acid
PSMA: prostate-specific membrane antigen
RP: reversed phase
RPMI: Roswell Park Memorial Institute
SD: standard deviation
SPECT: single photon emission computed tomography
TOF: time of flight
