# Supplementary material for: Development of a new class of PSMA radioligands comprising ibuprofen as an albumin-binding entity
Source: Theranostics. 2020 Jan 1;10(4):1678–93. doi: 10.7150/thno.40482 (PMC6993238; doi:10.7150/thno.40482)
Supplement: Supplementary file 1 — Supplementary methods, schemes, results, figures and tables. [file thnov10p1678s1.pdf]

SUPPLEMENTARY MATERIAL

Development of a new class of PSMA radioligands comprising ibuprofen as an albumin-binding entity

Luisa M. Deberle[1,2†], Martina Benešová[1,2†], Christoph A. Umbricht[2], Francesca Borgna[2], Manuel Büchler[2], Konstantin Zhernosekov[3], Roger Schibli[1,2], Cristina Müller[1,2*]

1. Department of Chemistry and Applied Biosciences, ETH Zurich, 8093 Zurich, Switzerland
2. Center for Radiopharmaceutical Sciences ETH-PSI-USZ, Paul Scherrer Institute, 5232 Villigen-PSI, Switzerland
3. Isotope Technologies GmbH Garching, 85748 Garching, Germany

E-Mail addresses:
luisa.deberle@psi.ch; martina.benesova@t-online.de; christoph.umbricht@psi.ch; francesca.borgna@psi.ch; buemanue@student.ethz.ch; konstantin.zhernosekov@itg-garching.de; roger.schibli@psi.ch; cristina.mueller@psi.ch
[†] equally contributed to this article

***Correspondence to**:
PD Dr. Cristina Müller
Center for Radiopharmaceutical Sciences ETH-PSI-USZ
Paul Scherrer Institut
5232 Villigen-PSI
Switzerland
e-mail: cristina.mueller@psi.ch
phone: +41-56-310 44 54; fax: +41-56-310 28 49

1. HPLC purification

High performance liquid chromatography (HPLC) of the four ibuprofen-derivatized PSMA ligands was performed with a Merck-Hitachi LaChrom HPLC system, equipped with a D-7000 interface, a L-7200 autosampler, a L-7400 UV detector, and a L-7100 pump. A reversed-phase C18 column (5 µm, 4.6 x 150 mm, Sunfire^{TM}, Waters, USA) was used for analytical purposes. Purification of the PSMA ligands was carried out using a semi-preparative reversed-phase C18 column (5 µm, 10×150 mm, Sunfire^{TM}, Waters, USA). In both cases, the mobile phase consisted of 0.1% (*v*/*v*) trifluoroacetic acid (TFA) in Milli-Q water (A) and acetonitrile (ACN) (B). A linear gradient of solvent A (95–5% over 15 min) in solvent B at a flow rate of 1 mL/min was used for analytical runs. A linear gradient of solvent A (90–50% over 20 min) in solvent B at a flow rate of 4 mL/min was used for preparative runs. The fractions containing the pure product were collected in a round-bottom flask, frozen in liquid nitrogen and lyophilized for ≥24 h to obtain the pure product as dried powder. Analytical data are presented in Table 1 of the main article.

2. Synthesis of PSMA ligands

The synthesis and purification of Ibu-N-PSMA and Ibu-DAB-PSMA was performed in analogy to the synthesis of Ibu-Dα-PSMA as described in the main article, however, instead of using an aspartate-based linker to conjugate (*RS*)-ibuprofen, D-asparagine and D-diaminobutyric acid-based linkers, respectively, were employed.

Methods: Synthesis of Ibu-N-PSMA (Scheme S1): The linker entity consisting of D-asparagine (D-Asn) was conjugated to *N*ε-L-lysine of the precursor **5** before coupling the (*RS*)-ibuprofen. The resin-immobilized precursor **5** was pre-swollen in dichloromethane (DCM) for 45 min and subsequently conditioned in *N*,*N*-dimethylformamide (DMF). Relative to precursor **5** (0.10 mmol), 4.0 equiv Fmoc-N-trityl-D-asparagine (Fmoc-D-Asn(Trt)-OH, 0.40 mmol) were activated using 3.96 equiv *O*-(benzotriazol-1-yl)-*N,N,N′,N′*-tetramethyluronium hexafluorophosphate (HBTU, 0.40 mmol) in the presence of 4.0 equiv *N,N*-diisopropylethylamine (DIPEA, 0.40 mmol) in anhydrous DMF. Two minutes after the addition of DIPEA, the activated solution was added to the precursor **5** and agitated for 3 h. The resin-immobilized compound **10** was washed with DMF and and the *N*α-Fmoc-protecting group was cleaved by agitating with a mixture of DMF and piperidine in a ratio of 1:1 (*v*/*v*) twice for 5 min to yield compound **11**. The resin-immobilized compound was again washed with DMF. A racemic mixture of 2-(4-(2-methylpropyl)phenyl)propanoic acid ((*RS*)-ibuprofen, 4.0–6.0 equiv; 0.40–0.60 mmol) was activated using 3.96 equiv HBTU (0.40–0.59 mmol) in the presence of 4.0–6.0 equiv DIPEA (0.40–0.60 mmol) in anhydrous DMF. Two minutes after the addition of DIPEA, the activated solution was added to the resin and agitated for 2 h to give compound **12** immobilized on the resin. It was washed with DMF, DCM and diethyl ether, respectively, and dried under reduced pressure. The product was cleaved from the resin with a mixture consisting of TFA, triisopropylsilane (TIPS) and

Milli-Q in a ratio of 95:2.5:2.5 (*v*/*v*) within 3–6 h. The *t*-Bu-protecting groups and the additional Trt-protecting group were cleaved simultaneously. TFA was evaporated, the crude compound dissolved in ACN and Milli-Q water in a ratio of 1:2 (*v*/*v*) and purified by HPLC to yield Ibu-N-PSMA.

Scheme S1. Synthesis of Ibu-N-PSMA.

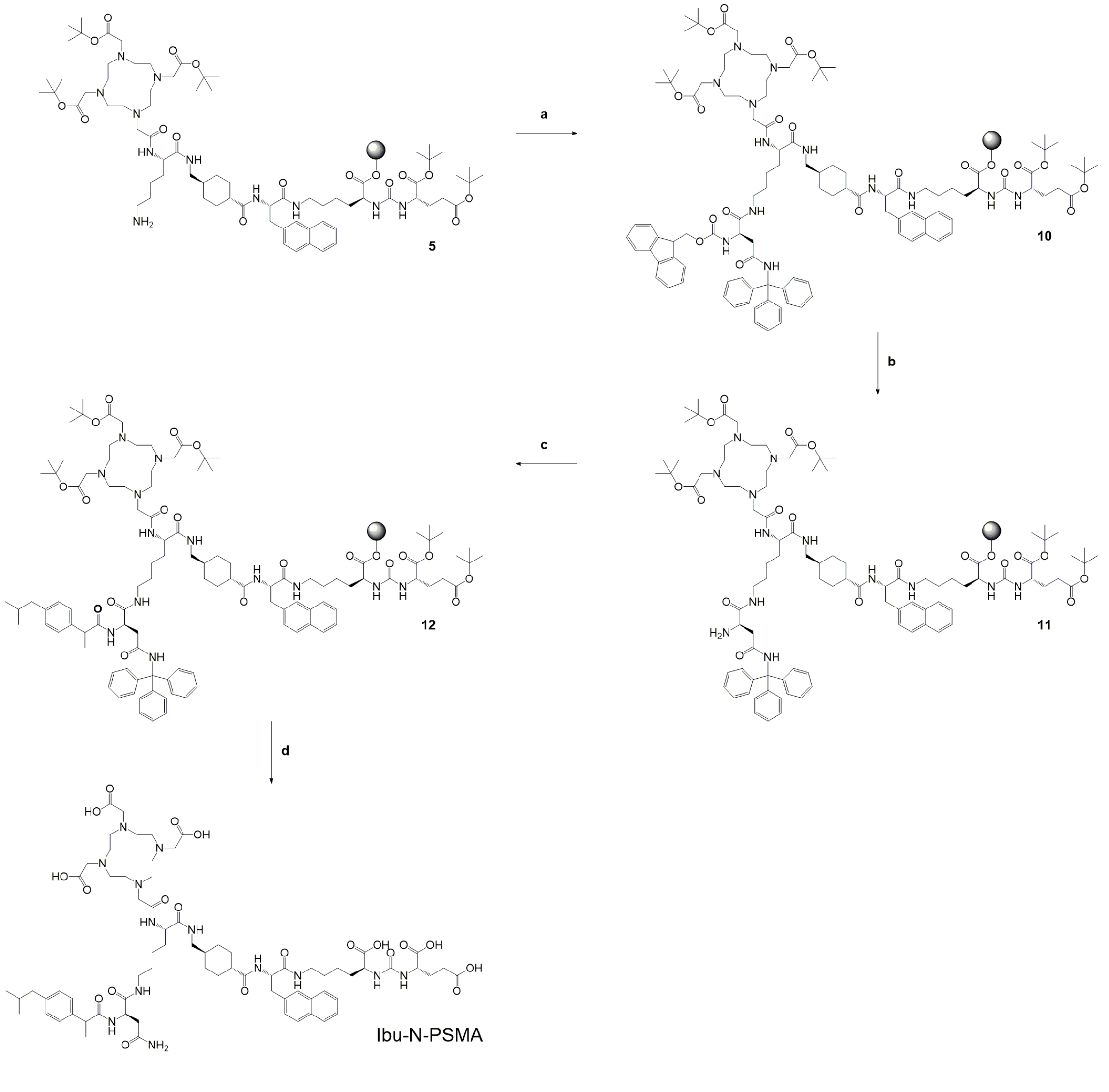

a) Fmoc-D-Asn(Trt)-OH, HBTU, DIPEA, DMF; b) 50% piperidine in DMF c) Ibuprofen, HBTU, DIPEA, DMF;
d) TFA, TIPS, H_2O 95:2.5:2.5

Methods: Synthesis of Ibu-DAB-PSMA (Scheme S2): The linker entity consisting of D-diaminobutyric acid was conjugated to *N*ε-L-lysine of the precursor **5** before coupling the (*RS*)-ibuprofen. The resin-immobilized precursor **5** was pre-swollen in DCM and conditioned in DMF as described above. Relative to precursor **5** (0.10 mmol), 4.0 equiv N_a-Fmoc-$N_ε$-boc-D-diaminobutyric acid (DAB; Fmoc-D-Dab(Boc)-OH 0.40 mmol) were activated using 3.96 equiv HBTU (0.40 mmol) in the presence of 4.0 equiv DIPEA (0.40 mmol) in anhydrous DMF. Two minutes after the addition of DIPEA, the activated solution was added to the precursor **5** and agitated for 3.5 h. The resin-immobilized compound **13** was washed with DMF and the *N*α-Fmoc-protecting group was cleaved by agitating with a mixture of DMF and piperidine in a ratio of 1:1 (*v*/*v*) twice for 5 min to give compound **14**. The resin-immobilized compound was again washed with DMF. (*RS*)-Ibuprofen (4.0–6.0 equiv; 0.40–0.60 mmol) was activated using 3.96 equiv HBTU (0.40–0.59 mmol) in the presence of 4.0–6.0 equiv DIPEA (0.40–0.60 mmol) in anhydrous DMF. Two minutes after the addition of DIPEA, the activated solution was added to the resin and agitated for 2 h to yield resin-immobilized compound **15**. It was washed with DMF, DCM and diethyl ether, respectively, and dried under reduced pressure. The product was cleaved from the resin with a mixture consisting of TFA, TIPS and Milli-Q water in a ratio of 95:2.5:2.5 (*v*/*v*) within 3–6 h. The *t*-Bu-protecting groups and the additional Boc-protecting group were cleaved simultaneously. TFA was evaporated, the crude compound dissolved in ACN and Milli-Q water in a ratio of 1:2 (*v*/*v*) and purified by HPLC to yield Ibu-DAB-PSMA.

Scheme S2. Synthesis of Ibu-DAB-PSMA.

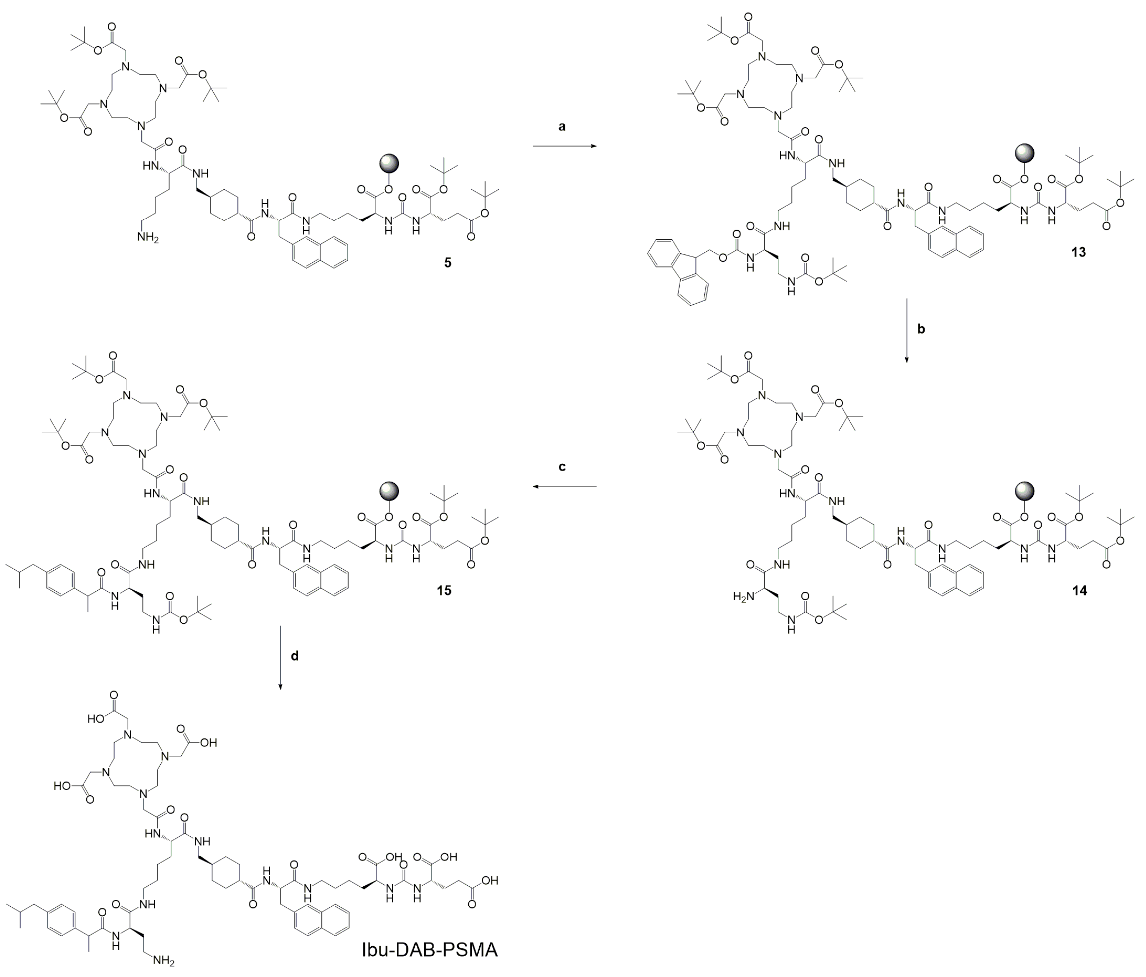

a) Fmoc-D-Dab(Boc)-OH, HBTU, DIPEA, DMF; b) 50% piperidine in DMF c) Ibuprofen, HBTU, DIPEA, DMF;
d) TFA, TIPS, H_2O 95:2.5:2.5

3. Radiolabeling of the PSMA ligands

Methods: The stock solution of PSMA-617 (ABX GmbH, Radeberg, Germany) was prepared by dilution of the ligand in Milli-Q water to a final concentration of 1 mM. Ibu-PSMA, Ibu-Dα-PSMA, Ibu-N-PSMA and Ibu-DAB-PSMA were diluted in Milli-Q water/sodium acetate (0.5 M, pH 8) to obtain a final concentration of 1 mM. The ligands were labeled with ^{177}Lu (no-carrier-added ^{177}Lu in 0.05 M HCl; Isotope Technologies Garching ITG GmbH, Germany) in a 1:5 (*v/v*) mixture of sodium acetate (0.5 M, pH 8) and HCl (0.05 M, pH ~1) at pH ~4.5 at a molar activity of commonly 50 MBq/nmol as previously reported for other ligands [1, 2]. The reaction mixture was incubated for 10 min at 95 °C, followed by a quality control using a Merck Hitachi LaChrom HPLC system, equipped with a D-7000 interface, a L-7200 autosampler, a radioactivity detector (LB 506 B; Berthold) and a L-7100 pump connected with a reversed-phase C18 column (Xterra™ MS, C18, 5 µm, 150 x 4.6 mm; Waters) in order to determine the radiochemical purity of the radioligands with regard to potential unreacted ^{177}Lu and radioactive species of unknown structure. The mobile phase consisted of Milli-Q water containing 0.1% TFA (A) and ACN (B). A linear gradient of solvent A (95–20% over 15 min) in solvent B at a flow rate of 1 mL/min was used. The radioligands were diluted in Milli-Q water containing sodium diethylenetriamine pentaacetic acid (Na-DTPA, 50 µM) prior to injection into HPLC.

Results: The quality control resulted in high radiochemical purity (≥96%) of the radioligands labeled at a molar activity of 50 MBq/nmol. Representative HPLC chromatograms of the ^{177}Lu-labeled PSMA ligands are shown in Figure S1.

Remark: radiolabeling at a molar activity up to 100 MBq/nmol was feasible. The results of the radiochemical purity of each radioligand labeled under these conditions are reported in the main article.

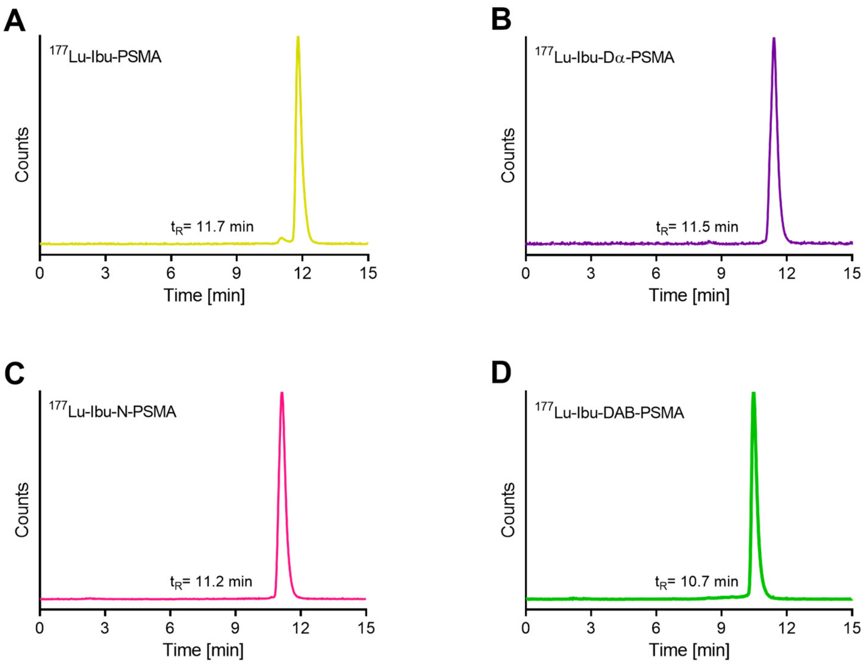

Figure S1. HPLC chromatograms of the ibuprofen-derivatized PSMA ligands after radiolabeling with ^{177}Lu (50 MBq/nmol). (A) Chromatogram of ^{177}Lu-Ibu-PSMA; **(B)** Chromatogram of ^{177}Lu-Ibu-Dα-PSMA; **(C)** Chromatogram of ^{177}Lu-Ibu-N-PSMA; **(D)** Chromatogram of ^{177}Lu-Ibu-DAB-PSMA. Retention times (t_R = 10.7—11.7 min) are indicated in the figures.

4. Identification of Lu-labeled ligands

Methods: The PSMA ligands were labeled with a two-fold excess of ^{175}Lu ($^{175}LuCl_3 \cdot 6\ H_2O$) in a 1:5 (*v/v*) mixture of sodium acetate (0.5 M, pH 8) and HCl (0.05 M, pH ~1) at pH ~4.5. The reaction mixture was incubated for 20 min at 95 °C, followed by addition of 35 µL of a 5 mM Na-DTPA solution. The analysis was performed using the same HPLC system (pump, autosampler and C-18 column) and HPLC program as was used for the active labeling. The chromatograms were, however, obtained at λ = 220 nm using the UV detector (Merck Hitachi LaChrom L-7400) to determine the retention times of each ^{175}Lu-labeled ligand. The ^{175}Lu-labeled ligands in the reaction mixture were identified using a Waters ACQUITY liquid chromatography−mass spectrometry (LC-MS) system equipped with a Sample Manager FTN, a Column Manager, a Quaternary Solvent Manager, a PDA Detector and a Waters SQ Detector 2.

Results: The quality control revealed high purity of the ligands. The signals, observed at the front of the chromatograms (t_R = 1.7 and 2.2 min), are artefacts as a consequence of the injection. The UV signals of the ligands were detected approximately 0.5 min before the corresponding radiosignal due to the arrangement of the HPLC device and respective dead times between UV detector and radiodetector. The

retention times of the UV signals of the ligands followed the same tendency as previously recorded for the radiosignals of the respective radioligands, with ^{175}Lu/^{177}Lu-Ibu-DAB-PSMA showing the shortest retention time followed by ^{175}Lu/^{177}Lu-Ibu-N-PSMA, ^{175}Lu/^{177}Lu-Ibu-Dα-PSMA and ^{175}Lu/^{177}Lu-Ibu-PSMA (Figure S2). LC-MS measurements confirmed the identity of the ^{175}Lu-labeled ligands (Table S1).

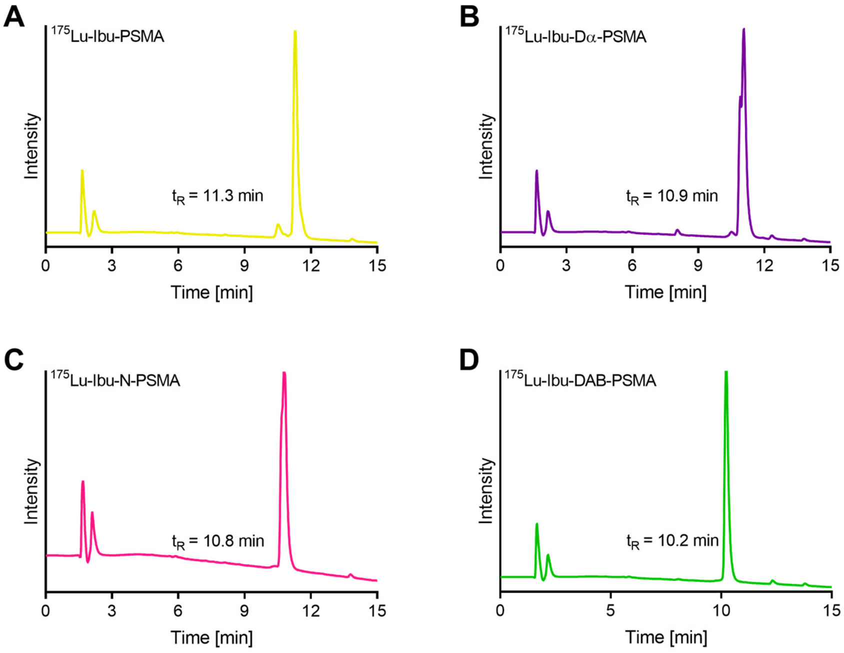

Figure S2. HPLC chromatograms of the ibuprofen-derivatized PSMA ligands after labeling with ^{175}Lu. (A) Chromatogram of ^{175}Lu-Ibu-PSMA; **(B)** Chromatogram of ^{175}Lu-Ibu-Dα-PSMA; **(C)** Chromatogram of ^{175}Lu-Ibu-N-PSMA; **(D)** Chromatogram of ^{175}Lu-Ibu-DAB-PSMA. Retention times (t_R = 10.2–11.3 min) are indicated in the figures.

Table S1. Found masses of the PSMA-ligands labeled with ^{175}Lu.

Compound	Calculated mass	Found mass[a]
^{175}Lu-Ibu-PSMA	1532.7	1532
^{175}Lu-Ibu-Dα-PSMA	1646.7	1646
^{175}Lu-Ibu-N-PSMA	1646.7	1646
^{175}Lu-Ibu-DAB-PSMA	1633.7	1632

[a] *m*/*z*-peak obtained by LC-MS detected as $[M + H]^+$;

5. Radiolytic stability

Methods: Radiolytic stability of each PSMA radioligand was assessed over a period of 24 h. For this purpose, Ibu-PSMA, Ibu-Dα-PSMA, Ibu-N-PSMA and Ibu-DAB-PSMA were labeled with ^{177}Lu in a volume of 120 μL at a molar activity of 50 MBq/nmol. After quality control using HPLC (t = 0, radiochemical purity ≥ 96%), the labeling solutions were diluted with saline to 250 MBq/500 μL and radioligand integrity was determined for each radioligand with and without addition of L-ascorbic acid (3 mg). The radioligand dilutions were incubated at room temperature and radioligand degradation was determined by HPLC after 1 h, 4 h and 24 h incubation time as previously reported [3]. The HPLC chromatograms were analyzed by integration of the peaks representing the radiolabeled product, the released ^{177}Lu as well as degradation products of unknown structure (Figure S3). A quantitative assessment was performed by expressing the peak area of the intact product as percentage of the sum of integrated peak areas of the entire chromatogram.

Results: The results are shown in Figure S3 and reported in the main article.

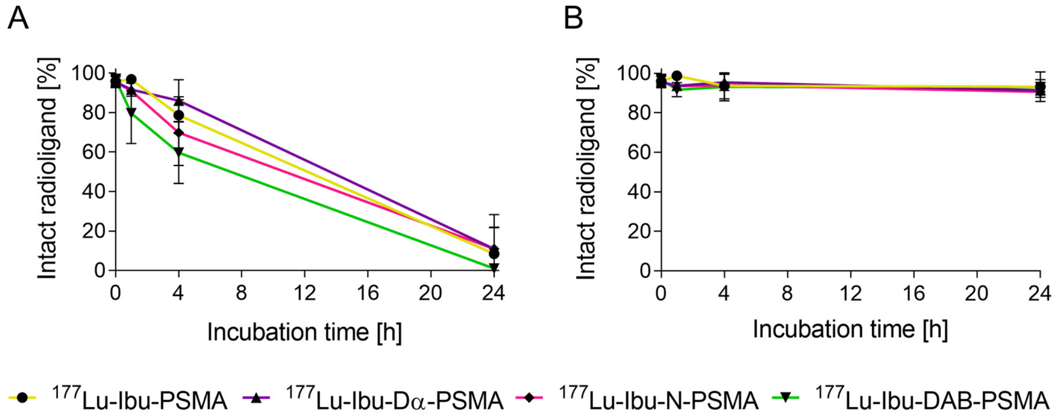

Figure S3. Radiolytic stability indicated as percentage of the intact radioligands up to 24 h. (A) Radioligands incubated in saline without L-ascorbic acid; **(B)** Radioligands incubated in saline containing L-ascorbic acid (average ± SD, n = 3).

6. *n*-Octanol/PBS distribution coefficient

Methods: The *n*-octanol/PBS distribution coefficients (logD values) of ^{177}Lu-Ibu-PSMA, ^{177}Lu-Ibu-Dα-PSMA, ^{177}Lu-Ibu-N-PSMA and ^{177}Lu-Ibu-DAB-PSMA were determined as previously reported [1]. The logD values were determined in a mixture of *n*-octanol and phosphate-buffered saline (PBS, pH 7.4) by a shake-flask method using liquid-liquid extraction followed by phase separation. The radioligands were labeled at 50 MBq/nmol and diluted in PBS (pH 7.4, ~500 kBq, 25 μL, 0.01 nmol). The radioligand dilution was then added to polypropylene tubes containing 1475 μL PBS and 1500 μL *n*-octanol and the mixtures were vortexed vigorously for 1 min. Phase separation was obtained by centrifugation at 1200 rcf for 6 min. The radioactivity in the PBS and *n*-octanol phase was measured in a γ-counter (Perkin Elmer, Wallac Wizard 1480) in order to calculate the distribution coefficients as the logarithm of the ratio of counts per minute (cpm) measured in the *n*-octanol phase relative to the cpm measured in the PBS phase. Three experiments were performed in quintuplicate for all four radioligands.

7. Cell culture

Methods: Sublines of the androgen-independent PC-3 human prostate cancer cell line derived from an advanced androgen-independent bone metastasis had been created by transfection to express PSMA at high levels (PSMA-positive PC-3 PIP cells) or by mock transfection to obtain a cell line, which does not express PSMA (PSMA-negative PC-3 flu cells) [4]. Both cell lines have been used extensively by different groups, including our own, to evaluate a series of PSMA-targeting radiopharmaceuticals [2, 5, 6]. Cells were grown in a humidified incubator at 37 °C and 5% CO_2 in RPMI-1640 cell culture medium supplemented with 10% fetal calf serum, L-glutamine, antibiotics, as well as puromycin (2 μg/mL) to maintain PSMA expression. Twice a week, cells were split using PBS/EDTA and trypsin for detachment in order to maintain the cell culture.

8. Cell uptake and internalization

Methods: Uptake and internalization of the herein presented radioligands in PC-3 PIP and PC-3 flu cells were determined as previously reported [1]. All PSMA ligands were radiolabeled with ^{177}Lu at a molar activity of 5 MBq/nmol and diluted to 1.5 MBq/mL. PC-3 PIP or PC-3 flu cells were seeded in 12-well plates (~3×10^5 cells in 2 mL standard growth medium/well) allowing adhesion and growth overnight. The cells were washed once with PBS, prior to the addition of RPMI-1640 cell culture medium (975 μL/well) and the corresponding radioligand (37.5 kBq, 25 μL, 7.5 pmol per well, diluted in saline containing 0.05% bovine serum albumin (BSA)). The final BSA concentration in the well plates was low (0.00125%), but necessary to prevent adherence of the radioligands to the material of well plates. The well-plates were incubated for 2 h and 4 h at 37 °C and 5% CO_2. To determine the uptake of radioligand, the cells were washed three times with ice-cold PBS. The internalized fraction was determined in cells, which were washed with ice-cold PBS, then incubated for 10 min with acidic

stripping buffer (0.05 M glycine buffer in 100 mM NaCl, pH 2.8) followed by an additional washing step with ice-cold PBS. All cell samples were lysed by addition of NaOH (1 M, 1 mL) to each well and measured in a γ-counter (Perkin Elmer, Wallac Wizard 1480). The protein concentration was determined for each sample using a Micro BCA Protein Assay kit (Pierce, Thermo Scientific) in order to standardize the measured activity (percentage of total added activity) to the amounts of proteins in each well. The experiments were performed three times in triplicate for each compound and condition.

Results: The uptake of all radioligands into PC-3 PIP tumor cells was comparable to ^{177}Lu-PSMA-617 after incubation for a period of 2 h or 4 h, respectively (Figure S4). The internalized fraction of ^{177}Lu-Ibu-PSMA was slightly higher than for ^{177}Lu-Ibu-Dα-PSMA, ^{177}Lu-Ibu-N-PSMA, ^{177}Lu-Ibu-DAB-PSMA and ^{177}Lu-PSMA-617, which were all in the same range (Figure S4A). The uptake of all radioligands in PC-3 flu tumor cells was <2% of total added activity after 4 h incubation, indicating that the uptake in PC-3 PIP cells was PSMA-specific (Figure S4B).

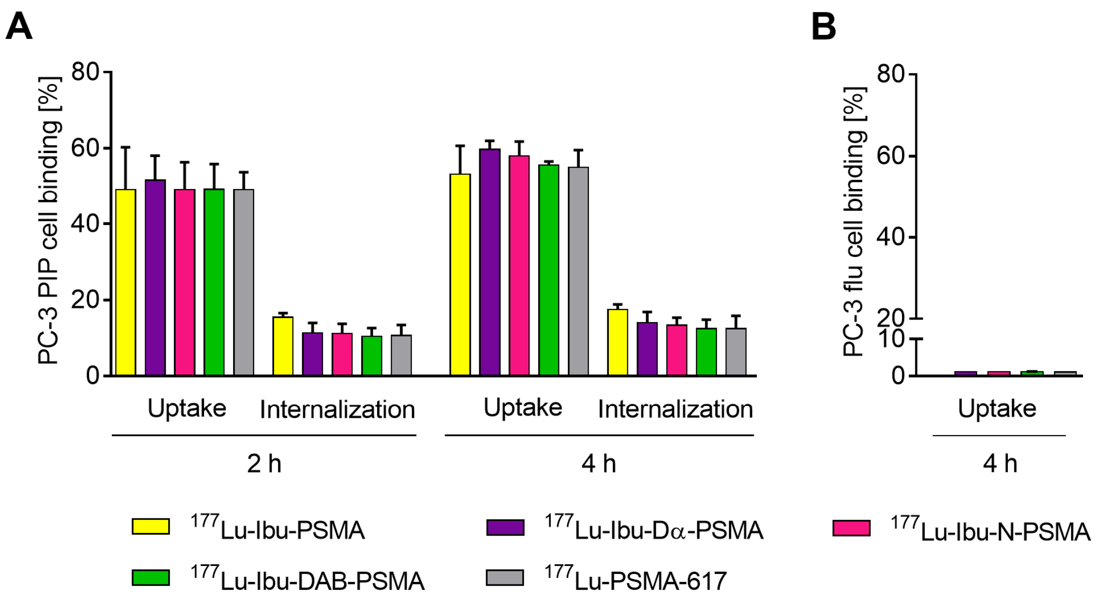

Figure S4. Uptake and internalization of ^{177}Lu-Ibu-PSMA, ^{177}Lu-Ibu-Dα-PSMA, ^{177}Lu-Ibu-N-PSMA and ^{177}Lu-Ibu-DAB-PSMA in comparison to ^{177}Lu-PSMA-617. (A) Data obtained with PSMA-positive PC-3 PIP cells (average ± SD, n=3). **(B)** Data obtained with PSMA-negative PC-3 flu cells (average ± SD, n=1).

9. SPECT/CT imaging studies

Methods: SPECT/CT experiments were performed using a dedicated small-animal SPECT/CT camera (NanoSPECT/CT^{TM}, Mediso Medical Imaging Systems, Budapest, Hungary) as previously reported [1, 2]. Scans were acquired 4 h and 24 h after injection of the radioligands. Each radioligand was labeled with ^{177}Lu at a molar activity of 25 MBq/nmol and diluted in saline containing 0.05% BSA. Mice were injected into a lateral tail vein with the respective radioligand (25 MBq, 1 nmol, 100 µL). Each SPECT scan lasted for about 45 min followed by a CT scan of 7.5 min, while the mice were anesthetized with a mixture of isoflurane and oxygen. Data reconstruction was performed using the NanoSPECT/CT™ software and images were prepared using the VivoQuant post-processing software (version 3.0, inviCRO Imaging Services and Software, Boston USA). A Gauss post-reconstruction filter (FWHM = 1 mm) was applied for all images and the scale of radioactivity was set as indicated on the scans (minimum value = 0.7 Bq/voxel to maximum value = 70 Bq/voxel).

The exact injected activity of ^{177}Lu-Ibu-PSMA was 21 MBq. At scan start after 4 h and 24 h, respectively, ~26% (5.4 MBq) and ~9.0% (1.9 MBq) of the injected activity was retained in the mouse body. A similar behavior was observed for ^{177}Lu-Ibu-Dα-PSMA (exact injected activity: 23 MBq). In this case ~29% (6.6 MBq) and 8.2% (1.9 MBq) were left at scan start after 4 h and 24 h p.i., respectively. The measurement of the mice injected with 24 MBq ^{177}Lu-Ibu-N-PSMA or 25 MBq ^{177}Lu-Ibu-DAB-PSMA revealed ~13% (3.2 MBq and 3.3 MBq, respectively) of the injected activity left after 4 h. At 24 h p.i., ~6.3% (1.5 MBq) and ~6.0% (1.5 MBq) of the injected activity was retained in the mice's bodies.

Results: The results are described in the main article.

10. Binding properties to mouse and human plasma proteins

Methods: The methods of the studies, performed to determine binding properties of the radioligands to mouse and human plasma proteins, are reported in the main article.

Results: The percentage of unspecific binding to the filter membrane, determined by incubation of the radioligands in PBS (pH 7.4) followed by filtration, was in the range of 0.4–8%. Whereas only a low fraction of ^{177}Lu-Ibu-PSMA, ^{177}Lu-Ibu-Dα-PSMA and ^{177}Lu-PSMA-617 was bound to the filter membrane, ^{177}Lu-Ibu-N-PSMA and ^{177}Lu-Ibu-DAB-PSMA showed a slightly higher and variable binding to the filter membrane. This might have led to a slight overestimation (≤8%) of the binding of these radioligands to mouse and human plasma proteins.

Table S2. Unspecific binding of the radioligands to the filter membrane.

Radioligand	Fraction bound to filter membrane [%]
^{177}Lu-Ibu-PSMA	2 ± 4
^{177}Lu-Ibu-Dα-PSMA	0.4 ± 0.9
^{177}Lu-Ibu-N-PSMA	4 ± 8
^{177}Lu-Ibu-DAB-PSMA	8 ± 9
^{177}Lu-PSMA-617	2 ± 1

11. Additional cell uptake and internalization experiments

Methods: A potential influence of the presence of BSA in the well-plates of an internalization experiment was investigated by additional studies with two of the radioligands: ^{177}Lu-Ibu-PSMA and ^{177}Lu-Ibu-DAB-PSMA. The radioligands were diluted with only saline or saline containing 0.05% BSA and applied (25 μL) to the well plates containing adherent PC-3 PIP cells to obtain final concentrations of 0% and 0.00125% BSA, respectively, in a total volume of 1 mL per well. The well-plates were incubated for 4 h at 37 °C. The experiments were performed three times in triplicate as reported above. Additional experiments were performed to evaluate a potential influence of an even higher concentration of BSA or human serum albumin (HSA) in the well-plates. ^{177}Lu-Ibu-PSMA and ^{177}Lu-Ibu-DAB-PSMA were diluted in 0.05% BSA, 0.5% BSA, 0.05% HSA or 0.5% HSA, resulting in 0.00125% and 0.0125% BSA or HSA in the wells. This experiment was performed once in triplicate as reported above.

Results: No difference in uptake and internalization was observed, irrespective of whether ^{177}Lu-Ibu-PSMA or ^{177}Lu-Ibu-DAB-PSMA were diluted in only saline or saline containing 0.05% BSA (Figure S5). Moreover, the use of BSA or HSA in different concentrations did not influence the

internalization and uptake of ^{177}Lu-Ibu-PSMA and ^{177}Lu-Ibu-DAB-PSMA, respectively (Figure S6). The results confirmed the data shown in Figure S4.

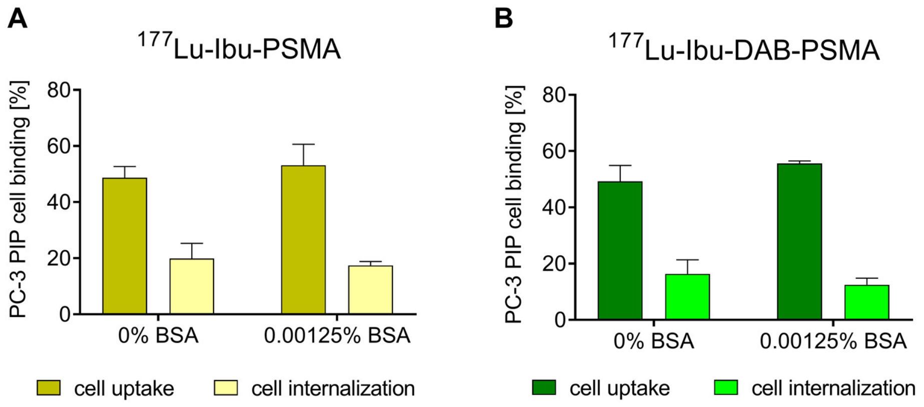

Figure S5. Uptake and internalization of radioligands into PC-3 PIP tumor cells applied on cells with 0% BSA or 0.00125% BSA. (A) ^{177}Lu-Ibu-PSMA (n=3); **(B)** ^{177}Lu-Ibu-DAB-PSMA (n=3). Data obtained with PSMA-positive PC-3 PIP cells.

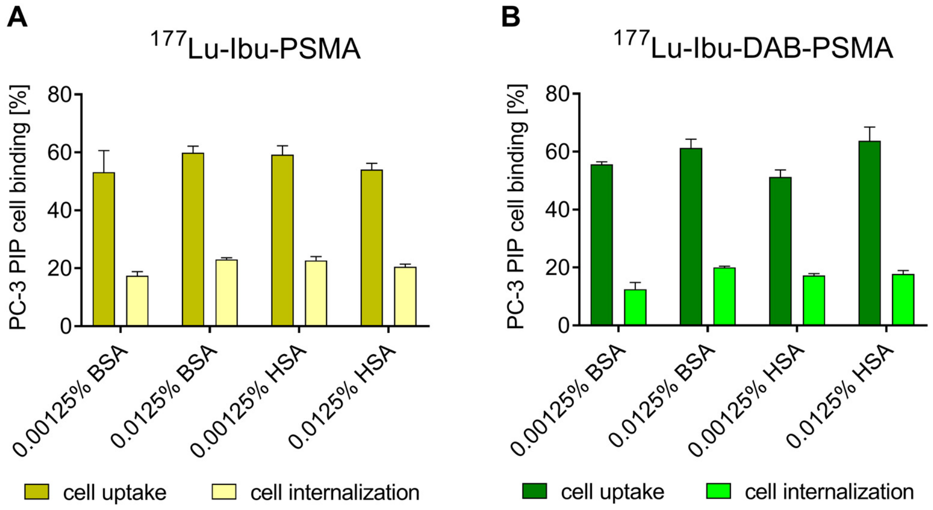

Figure S6. Uptake and internalization data of radioligands into PC-3 PIP tumor cells applied on cells with variable concentrations of BSA or HSA. (A) ^{177}Lu-Ibu-PSMA (n=1); **(B)** ^{177}Lu-Ibu-DAB-PSMA (n=1). Data obtained with PSMA-positive PC-3 PIP cells.

12. Biodistribution studies

Methods: The methods of the biodistribution studies are reported in the main article.

Results: The results were decay-corrected and listed as percentage of the injected activity per gram of tissue mass (% IA/g) (Table S3–S7). The most important biodistribution values of all ibuprofen-derivatized radioligands as well as of ^{177}Lu-PSMA-617 and ^{177}Lu-PSMA-ALB-56 are shown in Figure S7.

Table S3. Biodistribution data of ^{177}Lu-Ibu-PSMA in PC-3 PIP/flu tumor-bearing mice. The values are indicated as average ± SD obtained from each group of mice (n = 3−6) and listed as percentage of injected activity per gram tissue [% IA/g].

	**^{177}Lu-Ibu-PSMA**					
	**1 h p.i.**	**4 h p.i.**	**24 h p.i.**	**48 h p.i.**	**96 h p.i.**	**192 h p.i.**
Blood	15 ± 2	6.0 ± 1.5	0.59 ± 0.11	0.50 ± 0.02	0.36 ± 0.06	0.15 ± 0.05
Heart	5.7 ± 1.3	2.3 ± 0.7	0.32 ± 0.06	0.26 ± 0.02	0.15 ± 0.02	0.08 ± 0.02
Lung	13 ± 6	3.7 ± 0.8	0.59 ± 0.13	0.51 ± 0.08	0.30 ± 0.03	0.15 ± 0.03
Spleen	3.4 ± 0.4	1.8 ± 0.3	0.67 ± 0.13	0.50 ± 0.04	0.35 ± 0.05	0.25 ± 0.03
Kidneys	30 ± 4	33 ± 1	16 ± 3	11 ± 1	4.1 ± 0.3	2.2 ± 0.5
Stomach	2.2 ± 0.9	0.79 ± 0.26	0.21 ± 0.03	0.15 ± 0.00	0.15 ± 0.05	0.10 ± 0.07
Intestines	2.4 ± 0.3	1.0 ± 0.2	0.21 ± 0.05	0.14 ± 0.01	0.09 ± 0.02	0.04 ± 0.01
Liver	5.9 ± 0.4	2.8 ± 0.5	0.95 ± 0.11	0.85 ± 0.06	0.67 ± 0.07	0.50 ± 0.01
Salivary glands	4.2 ± 0.4	1.7 ± 0.4	0.32 ± 0.07	0.26 ± 0.02	0.14 ± 0.02	0.08 ± 0.02
Muscle	2.1 ± 0.4	0.97 ± 0.37	0.11 ± 0.04	0.09 ± 0.02	0.05 ± 0.01	0.03 ± 0.01
Bone	2.2 ± 0.3	1.0 ± 0.2	0.17 ± 0.05	0.13 ± 0.01	0.09 ± 0.01	0.06 ± 0.01
PC-3 PIP Tumor	49 ± 6	81 ± 7	77 ± 21	58 ± 4	28 ± 2	17 ± 4
PC-3 flu Tumor	4.4 ± 1.3	2.2 ± 0.6	0.60 ± 0.19	0.51 ± 0.15	0.18 ± 0.01	0.08 ± 0.02
Tumor-to-blood	3.2 ± 0.6	14 ± 3	133 ± 29	116 ± 10	79 ± 9	116 ± 26
Tumor-to-liver	8.2 ± 0.4	30 ± 4	82 ± 21	68 ± 8	42 ± 4	33 ± 5
Tumor-to-kidney	1.6 ± 0.1	2.6 ± 0.1	4.9 ± 0.7	5.5 ± 0.4	6.8 ± 0.4	7.7 ± 0.2

Table S4. Biodistribution data of ^{177}Lu-Ibu-Dα-PSMA in PC-3 PIP/flu tumor-bearing mice. The values are indicated as average ± SD obtained from each group of mice (*n* = 3−6) and listed as percentage of injected activity per gram tissue [% IA/g].

	**^{177}Lu-Ibu-Dα-PSMA**					
	**1 h p.i.**	**4 h p.i.**	**24 h p.i.**	**48 h p.i.**	**96 h p.i.**	**192 h p.i.**
Blood	18 ± 3	2.3 ± 0.8	0.33 ± 0.05	0.33 ± 0.13	0.27 ± 0.11	0.10 ± 0.03
Heart	5.4 ± 0.4	0.89 ± 0.27	0.17 ± 0.03	0.13 ± 0.04	0.11 ± 0.05	0.04 ± 0.01
Lung	10 ± 1	1.5 ± 0.4	0.37 ± 0.08	0.29 ± 0.06	0.22 ± 0.09	0.08 ± 0.02
Spleen	4.9 ± 0.9	0.90 ± 0.04	0.46 ± 0.09	0.33 ± 0.06	0.23 ± 0.04	0.15 ± 0.04
Kidneys	73 ± 2	27 ± 4	18 ± 3	7.2 ± 1.2	3.4 ± 0.4	1.1 ± 0.1
Stomach	1.7 ± 0.4	0.25 ± 0.08	0.14 ± 0.05	0.12 ± 0.04	0.06 ± 0.02	0.02 ± 0.00
Intestines	1.6 ± 0.4	0.49 ± 0.08	0.10 ± 0.02	0.07 ± 0.02	0.06 ± 0.02	0.03 ± 0.01
Liver	6.5 ± 0.1	0.84 ± 0.22	0.40 ± 0.05	0.28 ± 0.06	0.29 ± 0.08	0.13 ± 0.02
Salivary glands	4.6 ± 0.2	0.74 ± 0.20	0.19 ± 0.05	0.42 ± 0.55	0.09 ± 0.03	0.04 ± 0.00
Muscle	2.2 ± 0.2	0.35 ± 0.08	0.06 ± 0.02	0.04 ± 0.01	0.04 ± 0.01	0.01 ± 0.00
Bone	2.0 ± 0.1	0.37 ±0.10	0.10 ± 0.02	0.07 ± 0.01	0.05 ± 0.01	0.03 ± 0.00
PC-3 PIP Tumor	43 ± 5	49 ± 6	77 ± 15	49 ± 4	34 ± 5	17 ± 1
PC-3 flu Tumor	3.2 ± 0.4	1.0 ± 0.3	0.38 ± 0.03	0.20 ± 0.09	0.11 ± 0.02	0.04 ± 0.01
Tumor-to-blood	2.5 ± 0.4	23 ± 6	197 ± 27	174 ± 75	137 ± 49	193 ± 52
Tumor-to-liver	6.6 ± 0.4	61 ± 11	181 ± 42	186 ± 39	125 ± 34	132 ± 12
Tumor-to-kidney	0.59 ± 0.08	1.8 ± 0.1	3.5 ± 0.3	6.9 ± 0.7	10 ± 2	16 ± 2

Table S5. Biodistribution data of ^{177}Lu-Ibu-N-PSMA in PC-3 PIP/flu tumor-bearing mice. The values are indicated as average ± SD obtained from each group of mice (*n* = 3−6) and listed as percentage of injected activity per gram tissue [% IA/g].

	**^{177}Lu-Ibu-N-PSMA**					
	**1 h p.i.**	**4 h p.i.**	**24 h p.i.**	**48 h p.i.**	**96 h p.i.**	**192 h p.i.**
Blood	17 ± 3	3.6 ± 1.4	0.25 ± 0.07	0.25 ± 0.04	0.11 ± 0.04	0.08 ± 0.03
Heart	5.0 ± 0.9	1.2 ± 0.7	0.12 ± 0.03	0.20 ± 0.02	0.05 ± 0.02	0.04 ± 0.01
Lung	9.5 ± 1.2	2.2 ± 1.0	0.24 ± 0.06	0.27 ± 0.04	0.09 ± 0.03	0.07 ± 0.03
Spleen	3.6 ± 0.7	1.4 ± 0.8	0.24 ± 0.06	0.24 ± 0.05	0.08 ± 0.03	0.18 ± 0.04
Kidneys	33 ± 4	27 ± 9	8.0 ± 1.3	6.8 ± 1.3	2.4 ± 0.4	1.2 ± 0.2
Stomach	1.4 ± 0.7	0.51 ± 0.23	0.17 ± 0.09	0.22 ± 0.13	0.05 ± 0.01	0.07 ± 0.00
Intestines	1.9 ± 0.3	0.50 ± 0.18	0.09 ± 0.02	0.10 ± 0.04	0.03 ± 0.01	0.02 ± 0.00
Liver	3.6 ± 0.7	1.3 ± 0.6	0.32 ± 0.05	0.46 ± 0.05	0.08 ± 0.02	0.40 ± 0.04
Salivary glands	3.6 ± 0.5	0.94 ± 0.38	0.57 ± 0.35	0.10 ± 0.02	0.04 ± 0.01	0.03 ± 0.01
Muscle	1.9 ± 0.6	0.47 ± 0.19	0.06 ± 0.02	0.04 ± 0.01	0.02 ± 0.01	0.01 ± 0.00
Bone	2.0 ± 0.4	0.56 ± 0.22	0.08 ± 0.02	0.07 ± 0.02	0.04 ± 0.01	0.04 ± 0.01
PC-3 PIP Tumor	52 ± 3	65 ± 16	58 ± 21	52 ± 8	20 ± 3	16 ± 4
PC-3 flu Tumor	2.9 ± 0.7	1.2 ± 0.6	0.23 ± 0.01	0.15 ± 0.03	0.06 ± 0.01	0.04 ± 0.01
Tumor-to-blood	3.2 ± 0.3	21 ± 7	227 ± 42	208 ± 24	194 ± 38	204 ± 62
Tumor-to-liver	15 ± 2	18 ± 3	182 ± 53	114 ± 5	239 ± 25	39 ± 6
Tumor-to-kidney	1.6 ± 0.2	2.5 ± 0.2	6.9 ± 2.4	7.7 ± 0.6	8.3 ± 0.5	13 ± 2

Table S6. Biodistribution data of ^{177}Lu-Ibu-DAB-PSMA in PC-3 PIP/flu tumor-bearing mice. The values are indicated as average ± SD obtained from each group of mice (*n* = 3−6) and listed as percentage of injected activity per gram tissue [% IA/g].

	**^{177}Lu-Ibu-DAB-PSMA**					
	**1 h p.i.**	**4 h p.i.**	**24 h p.i.**	**48 h p.i.**	**96 h p.i.**	**192 h p.i.**
Blood	13 ± 4	3.7 ± 0.5	0.16 ± 0.02	0.10 ± 0.04	0.10 ± 0.04	0.06 ± 0.01
Heart	3.7 ± 1.1	1.3 ± 0.1	0.10 ± 0.01	0.06 ± 0.02	0.05 ± 0.01	0.03 ± 0.00
Lung	8.0 ± 1.7	2.4 ± 0.4	0.21 ± 0.03	0.14 ± 0.04	0.11 ± 0.02	0.08 ± 0.01
Spleen	2.9 ± 0.5	1.1 ± 0.1	0.32 ± 0.03	0.26 ± 0.08	0.19 ± 0.04	0.26 ± 0.03
Kidneys	30 ± 4	19 ± 2	6.0 ± 0.7	4.1 ± 0.9	2.3 ± 0.2	1.2 ± 0.2
Stomach	1.2 ± 0.7	0.62 ± 0.12	0.18 ± 0.13	0.06 ± 0.00	0.08 ± 0.05	0.03 ± 0.01
Intestines	1.6 ± 0.5	0.71 ± 0.05	0.11 ± 0.03	0.05 ± 0.01	0.04 ± 0.01	0.04 ± 0.03
Liver	3.1 ± 1.0	1.5 ± 0.2	0.56 ± 0.10	0.49 ± 0.08	0.24 ± 0.04	0.37 ± 0.02
Salivary glands	3.2 ± 1.1	0.86 ± 0.38	0.56 ± 0.37	0.09 ± 0.03	0.06 ± 0.01	0.05 ± 0.01
Muscle	1.9 ± 0.9	0.54 ± 0.09	0.05 ± 0.01	0.03 ± 0.01	0.02 ± 0.00	0.01 ± 0.00
Bone	1.6 ± 0.5	0.62 ± 0.08	0.09 ± 0.02	0.06 ± 0.01	0.04 ± 0.00	0.04 ± 0.00
PC-3 PIP Tumor	61 ± 10	66 ± 11	52 ± 3	36 ± 10	33 ± 2	27 ± 1
PC-3 flu Tumor	2.7 ± 0.7	1.2 ± 0.3	0.17 ± 0.01	0.15 ± 0.04	0.10 ± 0.02	0.08 ± 0.06
Tumor-to-blood	4.8 ± 0.8	18 ± 3	337 ± 40	364 ± 67	381 ± 115	492 ± 96
Tumor-to-liver	20 ± 3	45 ± 9	98 ± 21	72 ± 11	142 ± 15	74 ± 2
Tumor-to-kidney	2.0 ± 0.2	3.0 ± 0.5	8.9 ± 1.0	8.7 ± 1.8	15 ± 1	22 ± 2

Table S7. Biodistribution data 4 h after injection of ^{177}Lu-Ibu-PSMA diluted in saline or in saline containing 0.05% BSA in PC-3 PIP/flu tumor-bearing mice. The values are indicated as average ± SD obtained from each group of mice (*n* = 4) and listed as percentage of injected activity per gram tissue [% IA/g].

	**Saline**	**Saline with 0.05% BSA**
Blood	9.4 ± 1.4	6.0 ± 1.5
Heart	3.2 ± 0.6	2.3 ± 0.7
Lung	6.5 ± 1.1	3.7 ± 0.8
Spleen	2.6 ± 0.4	1.8 ± 0.3
Kidneys	36 ± 2	33 ± 1
Stomach	1.5 ± 0.7	0.79 ± 0.26
Intestines	1.2 ± 0.3	1.0 ± 0.2
Liver	4.0 ± 0.4	2.8 ± 0.5
Salivary glands	2.3 ± 0.3	1.7 ± 0.4
Muscle	1.0 ± 0.2	0.97 ± 0.37
Bone	1.2 ± 0.2	1.0 ± 0.2
PC-3 PIP Tumor	81 ± 12	81 ± 7
PC-3 flu Tumor	2.3 ± 0.3	2.2 ± 0.6
Tumor-to-blood	8.7 ± 1.4	14 ± 3
Tumor-to-liver	20 ± 4	30 ± 4
Tumor-to-kidney	2.2 ± 0.4	2.6 ± 0.1

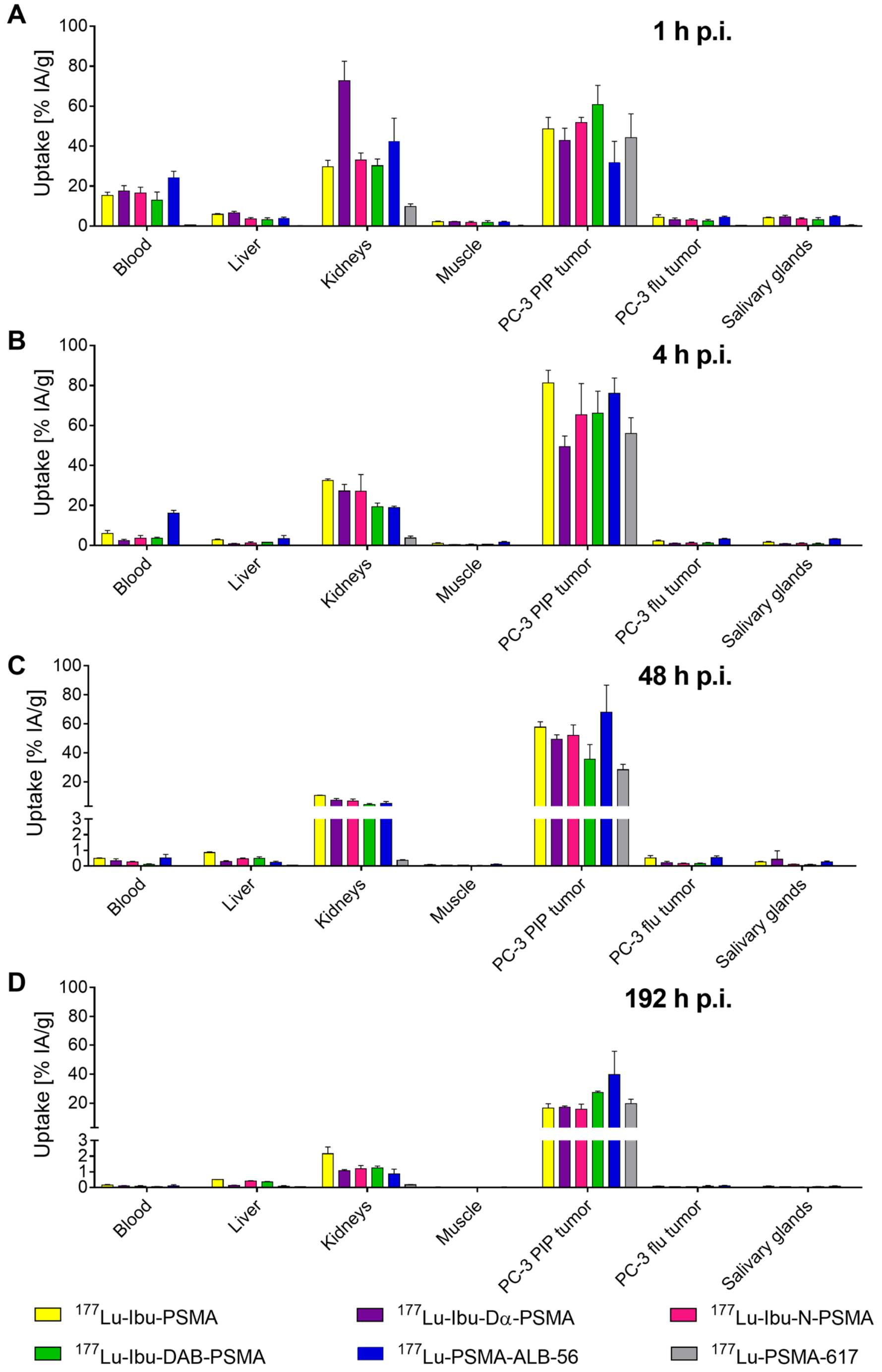

Figure S7. Biodistribution data of the ibuprofen-derivatized radioligands in PC-3 PIP/flu tumor-bearing mice. The decay-corrected accumulation (% IA/g-values) of the radioligands are presented for the most important organs and tissues. **(A)** Tissue uptake 1 h after injection; **(B)** Tissue uptake 4 h after injection; **(C)** Tissue uptake 48 h after injection and **(D)** Tissue uptake 192 h after injection of the radoligands. Data of ^{177}Lu-PSMA-ALB-56 [2] and ^{177}Lu-PSMA-617 [1] were included for comparison. Each data point represents the average of a group of mice ± SD (n = 3−6).

13. Area under the curves (AUC) values and AUC ratios

Methods: The areas under the curve (AUC) were calculated for the PC-3 PIP tumor, the blood, kidneys, liver and salivary glands based on non-decay corrected time-dependent biodistribution data.

Results: The results are shown in Figures 3 and 4 of the main article and listed in Table S8.

Table S8. Area under the curve (AUC) values and ratios of AUCs based on non-decay-corrected biodistribution data of the ibuprofen-bearing radioligands.

	^{177}Lu-Ibu-PSMA	^{177}Lu-Ibu-Dα-PSMA	^{177}Lu-Ibu-N-PSMA	^{177}Lu-Ibu-DAB-PSMA	^{177}Lu-PSMA-617*
**AUC values [% IA/g*h]**					
PC-3 PIP tumor	5896 ± 320	5569 ± 284	4770 ± 410	5187 ± 255	3691 ± 156
Blood	194 ± 16	144 ± 10	143 ± 15	129 ± 8	52 ± 2
Kidneys	1279 ± 38	1211 ± 61	868 ± 91	652 ± 29	99 ± 11
Liver	129 ± 6	55 ± 5	53 ± 6	68 ± 4	6.2 ± 1.6
Salivary glands	6.4 ± 0.4	5.9 ± 0.7	5.2 ± 0.5	4.7 ± 0.8	0.5 ± 0.2
**Ratios of AUC values**					
AUC_{Tu}-to-AUC_{Bl}	30 ± 5	39 ± 5	33 ± 7	40 ± 5	71 ± 6
AUC_{Tu}-to-AUC_{Ki}	4.6 ± 0.4	4.6 ± 0.5	5.5 ± 1.1	8.0 ± 0.8	37 ± 6
AUC_{Tu}-to-AUC_{Li}	46 ± 5	102 ± 13	90 ± 18	77 ± 9	592 ± 179
AUC_{Tu}-to-AUC_{SG}	915 ± 102	950 ± 158	922 ± 169	1108 ± 252	7382 ± 3265

* Data for ^{177}Lu-PSMA-617 were previously published by Benešová et al. 2018 [1]

14. Determination of the in vivo excretion of the radioligands

Methods: In order to determine the retention and excretion of each radioligand, non-tumor-bearing athymic BALB/c nude mice were injected with each radioligand (n = 2−3; per radioligand). The mice were measured for radioactivity immediately after injection and at several time points after injection up to 56 h p.i. (Figure S8). The PSMA ligands were, therefore, labeled at a molar activity of 25 MBq/nmol and diluted in saline containing 0.05% bovine serum albumin (BSA). Mice were intravenously injected with the respective radioligand (25 MBq, 1 nmol, 100 μL) and – after emptying the urinary bladder – measured in a dose calibrator immediately (t = 0) and at various time points after injection up to 56 h p.i.. The activity measured right after injection was set as 100%. The data points present the average of three mice per radioligand ± standard deviation (SD).

Results: All four novel radioligands showed a higher retention than PSMA-617, which can be ascribed to the albumin-binding properties of the incorporated ibuprofen. The ibuprofen-bearing radioligands exhibited, however, a faster excretion than the previously evaluated ^{177}Lu-PSMA-ALB-56 containing a *p*-tolyl moiety as stronger albumin binder [2]. The whole-body measurements revealed different excretion patterns for the single radioligands, which was manifested most prominently between 1 h and 4 h p.i. At all measured time points, retention was highest for the radioligand without additional linker entity, ^{177}Lu-Ibu-PSMA. At 1 h after injection, body retention of ^{177}Lu-Ibu-PSMA was 98%, compared to 73% (^{177}Lu-Ibu-Dα-PSMA and ^{177}Lu-Ibu-N-PSMA) and 62% (^{177}Lu-Ibu-DAB-PSMA).

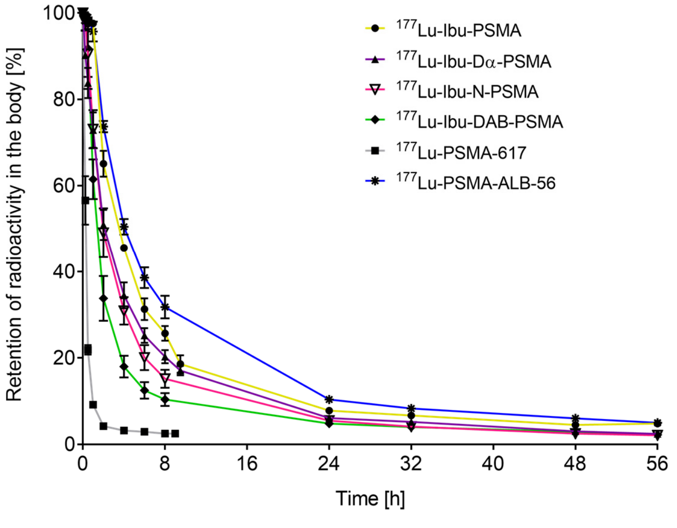

Figure S8. Whole-body activity of non-tumor-bearing mice up to 56 h after injection of the respective radioligands. The activity measured right after injection was set as 100%. Whole-body-activity measurement data of ^{177}Lu-PSMA-ALB-56 and ^{177}Lu-PSMA-617 was included in this graph for comparison. The data points present the average of two or three mice, which were injected with the same radioligand.

^{177}Lu-Ibu-DAB-PSMA was excreted fastest, with an activity retention of 18% at 4 h p.i., followed by ^{177}Lu-Ibu-N-PSMA (31%), ^{177}Lu-Ibu-Dα-PSMA (35%) and ^{177}Lu-Ibu-PSMA (46%). At this time point, ^{177}Lu-PSMA-617 was already almost entirely excreted (3.2%), while ^{177}Lu-PSMA-ALB-56 showed still 51% of activity in the body. In all cases of ibuprofen-derivatized radioligands, retention of activity in the body decreased over time and reached fractions of <5% at 48 h after injection.

References

1. Benešová M, Umbricht CA, Schibli R, Müller C. Albumin-binding PSMA ligands: optimization of the tissue distribution profile. Mol Pharm. 2018; 15: 934-46.
2. Umbricht CA, Benešová M, Schibli R, Müller C. Preclinical development of novel PSMA-targeting radioligands: modulation of albumin-binding properties to improve prostate cancer therapy. Mol Pharm. 2018; 15: 2297-306.
3. Siwowska K, Haller S, Bortoli F, Benešová M, Groehn V, Bernhardt P, et al. Preclinical comparison of albumin-binding radiofolates: impact of linker entities on the in vitro and in vivo properties. Mol Pharm. 2017; 14: 523-32.
4. Wu P, Kudrolli TA, Chowdhury WH, Liu MM, Rodriguez R, Lupold SE. Adenovirus targeting to prostate-specific membrane antigen through virus-displayed, semirandom peptide library screening. Cancer Res. 2010; 70: 9549-53.
5. Banerjee SR, Pullambhatla M, Foss CA, Nimmagadda S, Ferdani R, Anderson CJ, et al. ^{64}Cu-labeled inhibitors of prostate-specific membrane antigen for PET imaging of prostate cancer. J Med Chem. 2014; 57: 2657-69.
6. Choy CJ, Ling X, Geruntho JJ, Beyer SK, Latoche JD, Langton-Webster B, et al. ^{177}Lu-Labeled phosphoramidate-based PSMA inhibitors: the effect of an albumin binder on biodistribution and therapeutic efficacy in prostate tumor-bearing mice. Theranostics. 2017; 7: 1928-39.
